# *In silico* Accuracy and Energy Efficiency of Two Steering Paradigms in Directional Deep Brain Stimulation

**DOI:** 10.3389/fneur.2020.593798

**Published:** 2020-10-30

**Authors:** León Mauricio Juárez-Paz

**Affiliations:** Neuromodulation Research and Advanced Concepts, Boston Scientific Corporation, Valencia, CA, United States

**Keywords:** directional deep brain stimulation, stimulation steering, multiple independent current control, interleaving, Multi-Stim set, volume of tissue activated

## Abstract

**Background:** In Deep Brain Stimulation (DBS), stimulation field steering is used to achieve stimulation spatial specificity, which is critical to obtain clinical benefits and avoid side effects. Multiple Independent Current Control (MICC) and Interleaving/Multi Stim Set (Interleaving/MSS) are two stimulation field steering paradigms in commercially available DBS systems. This work investigates the stimulation field steering accuracy and energy efficiency of these two paradigms in directional DBS.

**Methods:** Volumes of Tissue Activated (VTAs) were generated *in silico* using pulse widths of 60 μs and five pulse amplitude fractionalizations intended to steer the VTAs radially in 12° steps. For each fractionalization, VTAs were generated with nine pre-defined target radii. Stimulation field steering accuracy was assessed based on the VTAs rotation angle. Energy efficiency was inferred from current draw from battery values, which were calculated based on the pulse amplitudes needed to generate and steer the VTAs, as well as electrode impedance measurements of clinically implanted directional leads.

**Results:** For radial steering, MICC needed a single VTA. In contrast, Interleaving/MSS required the generation of two VTAs, whose union and intersection created an Interleaving/MSS VTA and an Intersection VTA, respectively. MICC VTAs were 6.8 (−3.2–11.8)% larger than Interleaving/MSS VTAs. The Intersection VTAs accounted for 26.2 (16.0–32.8)% of Interleaving/MSS VTAs and were exposed to a higher stimulation frequency. For all VTA radius-fractionalization combinations, steering accuracy was 7.0 (4.5–10.5)° for MICC and 24.0 (9.0–25.3)° for Interleaving/MSS. Pulse amplitudes were 16.1 (9.2–28.6)% lower for MICC than for Interleaving/MSS, leading to a 45.9 (18.8–72.6)% lower current draw from battery for MICC.

**Conclusions:** The results of this work show that *in silico*, MICC achieves a significantly better stimulation field steering accuracy and has a significantly higher energy efficiency than Interleaving/MSS. Although direct evidence still needs to be generated to translate the results of this work to clinical practice, clinical outcomes may profit from the better stimulation field steering accuracy of MICC and longevity of DBS systems may profit from its higher energy efficiency.

## Introduction

Clinical studies investigating Deep Brain Stimulation (DBS) have provided evidence of its effectiveness in the treatment of motor symptoms in movement disorders such as Parkinson's Disease (PD) and Dystonia (1). Deep Brain Stimulation involves stimulation of specific brain structures by means of electrical pulses with a defined amplitude, width, and frequency. Pulses are generated by an Implantable Pulse Generator (IPG) connected through implanted electrical wires to an array of electrodes targeted to be placed adjacent to specific brain structures. The electrodes in the array could have a ring shape or be segmented (i.e., directional), with the latter having a smaller radial span that allows delivering more focal stimulation that results in clinical benefits (2–6). However, the use of directional leads in DBS involves new challenges in the implant procedure, as the final orientation of the directional leads often deviates with the intended orientation (7). Therefore, the accuracy to steer stimulation fields, which depends on the electronic architecture of the IPG, play an important role in directional DBS.

Commercially available DBS systems use either voltage controlled or current controlled electronic architectures. Voltage controlled systems set a fixed voltage at the stimulating electrodes, whereas current controlled systems set a fixed current flowing out of them (8). These two architectures could incorporate either a single source or multiple sources to generate the pulses. Single source architectures can deliver stimulation by activating one electrode or multiple electrodes simultaneously. In the latter case, referred to as coactivation (9), the pulse amplitude controlled by the single source will be distributed proportionally depending on the ratio of the impedances of the activated electrodes. Therefore, for coactivation, more current will flow through the electrodes with lower impedances. Multiple source architectures can explicitly specify the pulse amplitude delivered independently by each of the simultaneously activated electrodes. This architecture, combined with a current controlled architecture, ensures that the total current delivered to each electrode will remain constant regardless of change either in the total electrode impedance or the impedance ratio between the active electrodes. This ability enables a controlled steering of stimulation fields in DBS (10). The Multiple Independent Current Control technology (MICC) is an example of the combination of the multiple source and current controlled architectures.

Commercial stimulators, with either single source or multiple source architectures, also allow the control of the stimulation timing by delivering more than one pulse train through a lead or electrode. Historically, this ability in DBS has been referred to as Interleaving (11) and more recently to as Multi-Stim Set (MSS) stimulation (9). Interleaving/MSS involves the alternate, and hence not simultaneous, activation of single electrodes with a defined pulse amplitude (either voltage or current), which results in the alternating (staggering) generation of multiple stimulation fields. In the intersection of these stimulation fields, neural tissue will be stimulated with a higher frequency than outside the intersection (12). Interleaving/MSS has been suggested as a stimulation field steering option because it allows a temporal distribution of pulse amplitude (i.e., temporal fractionalization) between adjacent electrodes, which could aid single source systems to deliver pulses with a user-prescribed amplitude through multiple electrodes (9). To achieve this, Interleaving/MSS involves the manual titration of the pulse amplitude for each of the alternately activated electrodes to shape the volume result of the combination of the alternately generated stimulation fields (13).

For more than a decade, computational models have been used as a tool to estimate the amount of neuronal tissue surrounding DBS electrodes that gets activated by electrical stimulation (14). These computational models, referred to as Volume of Tissue Activated (VTA), generate three-dimensional representations of neural activation, which have been suggested as a tool to aid physicians in the clinical programming of DBS systems (15). Recently, computationally generated (i.e., *in silico*) VTAs have been incorporated into commercially available software (16), allowing an intuitive understanding of the effects of DBS in clinical practice, and a reduction in the time needed to program DBS settings (17–19). Moreover, these models have been used to generate probabilistic stimulation maps that aim to define brain regions with higher probability of producing good therapeutic outcomes for DBS (20, 21) and also to characterize the performance of directional stimulation for stimulation paradigms used in commercially available DBS systems (9). Based on computational models, this work compares the accuracy of MICC and Interleaving/MSS to steer (i.e., sculpt or shape) stimulation fields in directional DBS, and the energy efficiency associated to each of these paradigms. This comparison should contribute to clarifying the differences between MICC and Interleaving/MSS in directional DBS by characterizing, within a theoretical framework, their effectiveness, efficiency and limitations in the steering of stimulation fields.

## Methods

### Generation of VTAs

**Figure 1:**
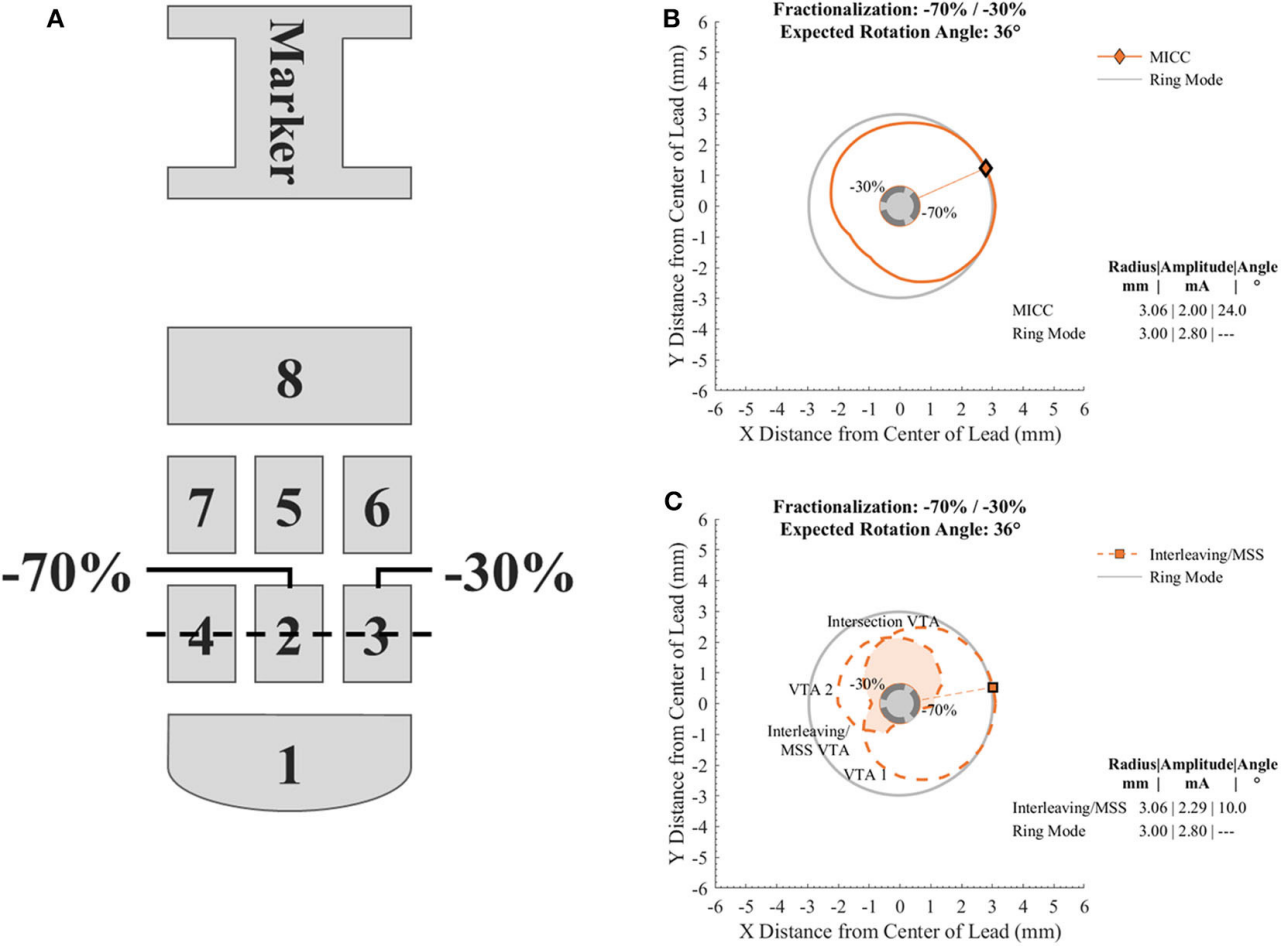
Diagram of the electrode array of the directional lead used to generate the VTAs and cross sections of VTAs generated with a target radius of 3.00 mm and a fractionalization of −70%/−30%. **(A)** Electrode nomenclature relative to the orientation marker on the top of the lead. For the case shown, electrodes 2 and 3 were used for the generation of the VTAs with a pulse amplitude distribution of −70 and −30%, respectively. The dotted line across electrodes 2, 3, and 4 indicates the level at which cross sections of VTAs were analyzed. **(B)** Cross section of a VTA generated for MICC. **(C)** Cross section of VTAs generated for Interleaving/MSS. VTA 1 and VTA 2 are VTAs generated for each of the activated electrodes. The union and intersection of VTA 1 and VTA 2 create the Interleaving/MSS VTA and Intersection VTA, respectively. The Intersection VTA is exposed to a higher stimulation frequency than the rest of the Interleaving/MSS VTA. Markers and radial lines indicate the radii and rotation angles of the VTAs.

Considering a current controlled electronic architecture, the *in silico* generation of VTAs was done for the Boston Scientific directional lead model 2202 (Figure 1A) using a customized MATLAB implementation of Boston Scientific's commercially available VTA simulation software (16), which is similar to earlier proposed software (14). VTAs were generated to have nine defined target radii (from 2.00 to 4.00 mm in 0.25 mm increments) for the cross section at the vertical center of the activated electrodes (Figure 1A dashed line) by simulating cathodic stimulation at 60 μs pulse width. The VTA radius for MICC and Interleaving/MSS was defined as the VTA's maximum radius at the angle that divided the cross section into two parts with equal areas (Figures 1B,C markers and radial lines). The angle of the line dividing the cross section of the VTA into two equal parts was found by rotating a straight line centered at the lead's axis in 1° steps. This angle defined the VTA rotation angle.

For all analyzed pulse amplitude distributions (Table 1), the total pulse amplitude was adjusted so that the maximum radius of the VTAs for MICC and Interleaving/MSS, reached a pre-defined VTA target radius (Figures 1B,C). Ring mode involves the activation of three electrodes, whereas single electrode activation only involves the activation of one electrode. Therefore, the term “fractionalizations” will be used for pulse amplitude distributions involving the activation of two electrodes (Table 1 pulse amplitude distributions 2–6).

**Table 1:** Pulse amplitude distributions used to generate the VTAs and their associated expected rotation angle.

**Pulse amplitude distributions**	**1**	**2**	**3**	**4**	**5**	**6**	**7**
Electrode 2	−34%	−50%	−60%	−70%	−80%	−90%	−100%
Electrode 3	−33%	−50%	−40%	−30%	−20%	−10%	0%
Electrode 4	−33%	0%	0%	0%	0%	0%	0%
Expected^a^ VTA angle rotation	-----	60°	48°	36°	24°	12°	0°

*Pulse amplitude distribution 1 corresponds to a ring mode stimulation configuration, whereas pulse amplitude distribution 7 corresponds to a single electrode activation configuration. The remaining pulse amplitude distributions involve two electrodes and will be referred to as fractionalizations. Pulse amplitude distributions 1 and 2 can be performed by means of coactivation in a single source system*.

a *By linear proportion*.

In the case of Interleaving/MSS, two VTAs (VTA 1 and VTA 2) were generated as a result of the individual activation of two neighboring electrodes (Table 1 electrode 2 and electrode 3, respectively). These two electrodes were activated with pulse amplitudes whose ratio corresponded to the analyzed fractionalizations. An Interleaving/MSS VTA and an Intersection VTA were created by the union and intersection, respectively, of VTA 1 and VTA 2 (Figure 1C).

The core elements of the computational model used to generate VTAs in this work have been previously described (10, 22). Briefly, a finite element model (FEM) was created to calculate the spatial distribution of the electric potential around the directional lead by simulating the lead and its surrounding medium. The encapsulation layer was set to 500 μm thickness and to a conductivity of 0.1 S/m, whereas for the bulk tissue, the conductivity was set to 0.2 S/m. This FEM model was coupled to a collection of myelinated axons modeled to have a diameter of 5.7 μm (MRG Model). These axons were oriented perpendicular to the lead's shaft and organized in two-dimensional matrices with a spacing of 0.5 mm, which were rotated in 30° steps. The neural response of these axons was modeled as described in (23). Finally, VTAs were generated by defining the activation threshold as the stimulation amplitude eliciting action potentials.

### Characterization of VTAs

Because this works aims to compare the accuracy of MICC and Interleaving/MSS for the radial steering of stimulation fields in directional DBS, the generated VTAs were characterized by quantifying their rotation angle. This angle was compared to each of the expected rotation angles for each fractionalization (Table 1) to quantify its deviation. For each fractionalization, steering accuracy was defined as the deviation from the expected rotation angle, being the first inversely proportional to the latter. The volume of the VTAs was calculated by summing the volume of their voxels. In the case of Interleaving/MSS, the calculations of the volumes for Interleaving/MSS VTA and Intersection VTA were based on the union and intersection, respectively, of VTA 1 and VTA 2. Additionally, for each fractionalization, the percentage of ring mode VTAs that MICC and Interleaving/MSS VTAs covered was calculated.

### Calculation of Energy Efficiency

The energy efficiency of the stimulation settings for each of the generated VTAs was characterized by quantifying their pulse amplitudes and current draw from battery. Pulse amplitudes were adjusted for each MICC and Interleaving/MSS fractionalization to generate VTAs with defined target radii. Current draw from battery was calculated considering a pulse frequency of 130 Hz and using equations previously reported (9). Equations (1), (3), and (4) describe the current draw from battery for MICC, Interleaving/MSS and coactivation, respectively. Current draw from battery was calculated for scenarios with equal and clinically measured electrode impedances. The scenario with equal electrode impedances considered an impedance of 3 kΩ. The scenario with clinically measured electrode impedances included a set of 980 measurements from directional electrodes of 24 clinically implanted leads. These impedance measurements were collected within the framework of the DIRECT DBS clinical study (Clinical Trials NCT02835274) from 12 subjects at seven study visits within a time period of 1 year. This collection allowed to capture realistic values for both impedance changes through time and impedance difference between neighboring electrodes. Current draw from battery is directly proportional to the pulse amplitude and impedance of each of the activated electrodes. Therefore, for the current draw from battery calculations, electrodes belonging to a same directional level in the same lead were permuted to form all possible pairs. This operation resulted in 1958 permutations for fractionalizations and 978 permutations for ring mode settings.

### Statistical Analyses

Comparing the MICC and Interleaving/MSS VTAs generated for each fractionalization, the null hypothesis is that their radii will be identical. Therefore, these radii were compared using a two-sided Wilcoxon signed-rank-test. In the case of VTA volumes, the null hypothesis is that for each fractionalization, MICC VTAs will be larger than Interleaving/MSS VTAs. For the steering accuracy, the null hypothesis is that MICC will generate VTAs with a higher accuracy (i.e., lower deviation from the expected rotation angle) than Interleaving/MSS for each fractionalization. Therefore, for each fractionalization, a one-sided Wilcoxon signed-rank-test was used for the comparison of MICC and Interleaving/MSS VTA volumes and steering accuracy across all analyzed VTA target radii.

For each of the analyzed VTA target radius, the null hypothesis is that MICC requires lower pulse amplitudes and lower current draw from battery than Interleaving/MSS. Additionally, the null hypothesis that coactivation requires lower pulse amplitudes and lower current draw from battery than MICC was tested. Therefore, a one-sided Wilcoxon signed-rank-test was also used for these comparisons. In the case of current draw from battery, this test was only used for the equal electrode impedance scenario. The choice for the Wilcoxon signed-rank-test was based on the small sample sizes for each of the analyzed VTA target radii and fractionalizations.

Because of the larger sample sizes and the normal distribution of the electrode impedance values (Kolmogorov-Smirnov-test, *p* < 0.001), a paired-sample *t*-test was used to compare the current draw from battery in the scenario with clinically measured electrode impedances. For this scenario, the comparison tested the same null hypotheses as in the scenario with equal electrode impedances. Moreover, the comparison was done for each of the VTA target radii and each fractionalization.

The data and MATLAB scripts used in this work will be made available to other researchers in accordance with the Boston Scientific Data Sharing Policy.

(1) $$\begin{array}{r} I_{MICC}=I_{overhead}\left(f\right)+\left(\overset{N}{\underset{i=1}{\sum }}I_{Ei}*PW*f*\frac{V_{\max}}{V_{bat}}\right) \end{array}$$

Where:

I_MICC_: Current draw from battery for MICC

I_overhead_(f): Frequency-dependent IPG overhead current, which was set to 4.9 μA

N: Number of activated electrodes

I_Ei_: Pulse amplitude for electrode i

PW: Pulse width, which was set to 60 μS

f: Pulse frequency, which was set to 130 Hz

V_max_: Maximum voltage for the activated electrodes (Equation 2)

V_bat_: Battery voltage, which was set to 2.8 V

(2) $$\begin{array}{r} V_{max}=max\left\{\left(I_{Ei}*Z_{Ei}\right):i=1..N\right\} \end{array}$$

Where:

V_max_: Maximum voltage for the activated electrodes

I_Ei_: Pulse amplitude for electrode i

Z_Ei_: Impedance of electrode i

N: Number of activated electrodes

(3) $$\begin{array}{r} I_{Interleaving/MSS}=I_{overhead}\left(N*f\right)+\overset{N}{\underset{i=1}{\sum }}\left(I_{Ei}*PW*f*\frac{V_{Ei}}{V_{bat}}\right) \end{array}$$

Where:

I_Interleaving/MSS_: Current draw from battery for Interleaving/MSS

I_overhead_(N^*^f): Frequency-dependent IPG overhead current, which was set to N^*^4.9 μA

N: Number of activated electrodes

I_Ei_: Pulse amplitude for electrode i

PW: Pulse width, which was set to 60 μS

f: Pulse frequency, which was set to 130 Hz

V_Ei_: Voltage for electrode i

V_bat_: Battery voltage, which was set to 2.8 V

(4) $$\begin{array}{r} I_{Coactivation}=I_{overhead}\left(f\right)+I_{total}*PW*f*\frac{V_{eq}}{V_{bat}} \end{array}$$

Where:

I_Coactivation_: Current draw from battery for Coactivation

I_overhead_(f): Frequency-dependent IPG overhead current, which was set to 4.9 μA

I_total_: Total stimulation pulse amplitude

PW: Pulse width, which was set to 60 μS

f: Pulse frequency, which was set to 130 Hz

V_eq_: Equivalent voltage for the contact configuration (Equation 5)

V_bat_: Battery voltage, which was set to 2.8 V

(5) $$\begin{array}{r} V_{eq}=\;\frac{I_{total}}{\sum_{i=1}^{N}\frac{1}{Z_{Ei}}} \end{array}$$

Where:

V_eq_: Equivalent voltage for the contact configuration

I_total_: Total stimulation pulse amplitude

Z_Ei_: Impedance of electrode i

N: Number of activated electrodes.

## Results

**Figure 2:**
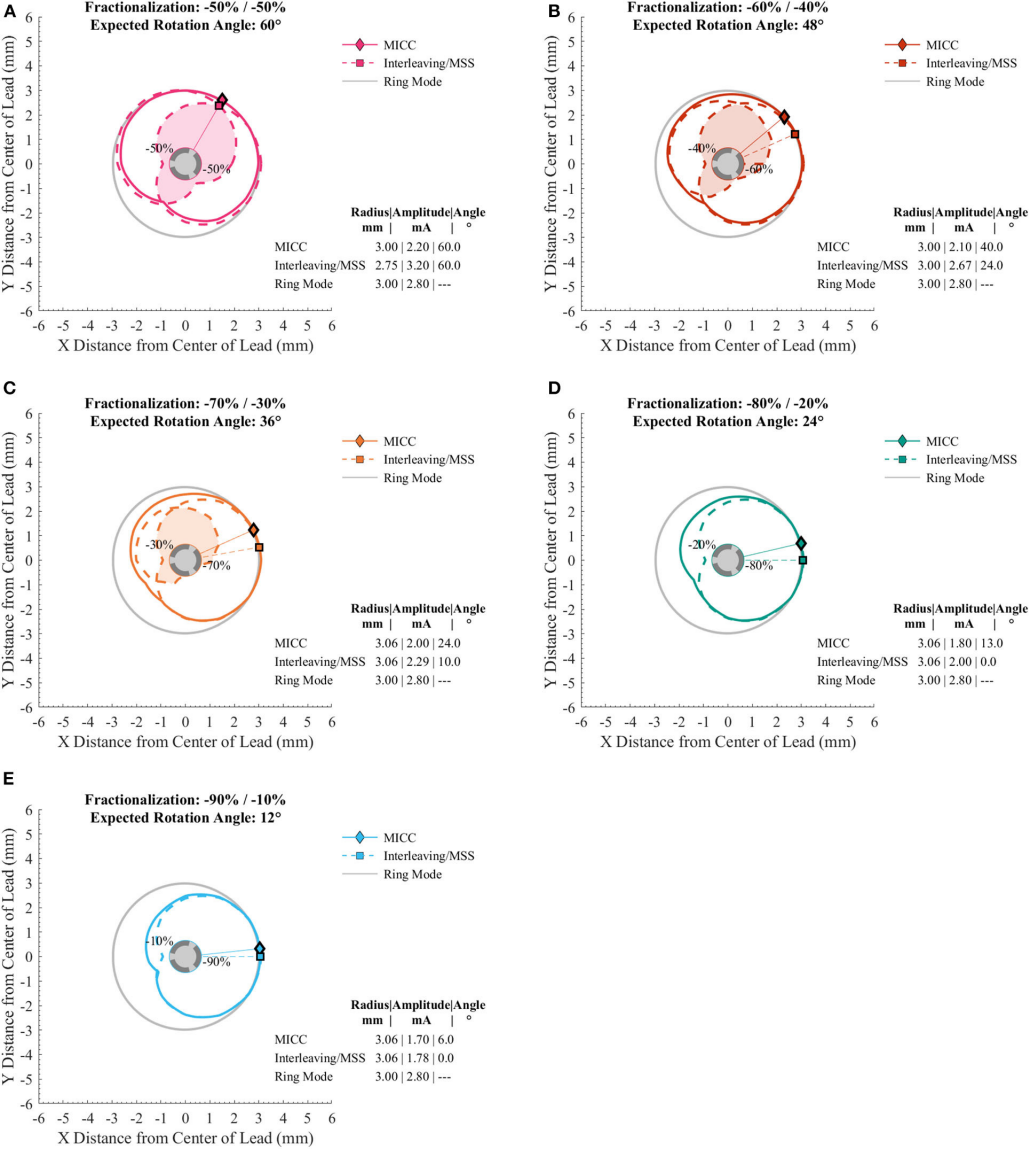
Cross sections of VTAs generated for MICC and Interleaving/MSS with a target radius of 3.00 mm. **(A–E)** Cross section of the VTAs generated for different MICC fractionalizations (solid lines), and the VTA 1 and VTA 2 (dashed lines). The filled area on the cross sections indicates the Intersection VTA, which is exposed to higher stimulation frequency, than outside this region. **(D,E)** Interleaving/MSS failed to produce the VTA for the electrode with the lower pulse amplitude (i.e., VTA 2). In all panels, markers and radial lines show the radii and rotation angles of the VTAs for MICC (rhombi) and Interleaving/MSS (squares).

VTAs were generated for different target radii (from 2.00 to 4.00 mm in 0.25 mm increments) and for different pulse amplitude distributions (Table 1) for MICC and Interleaving/MSS (Supplementary Figures 1A,B–9A,B). For MICC, a single VTA was generated for each VTA radius-fractionalization combination (Figure 2 solid lines). For Interleaving/MSS, VTA 1 and VTA 2 were generated for each of the activated electrodes and their union created the Interleaving/MSS VTA (Figure 2 dashed lines). In the cases where it was possible to generate both VTA 1 and VTA 2, these VTAs intersected creating an Intersection VTA (Figures 1C, 2 colored filled areas). In the scenario of equal electrode impedances, the generated VTAs for MICC and coactivation (i.e., ring mode and −50/−50% fractionalization) were identical due to the use of a homogeneous brain model for the simulations.

**Figure 3:**
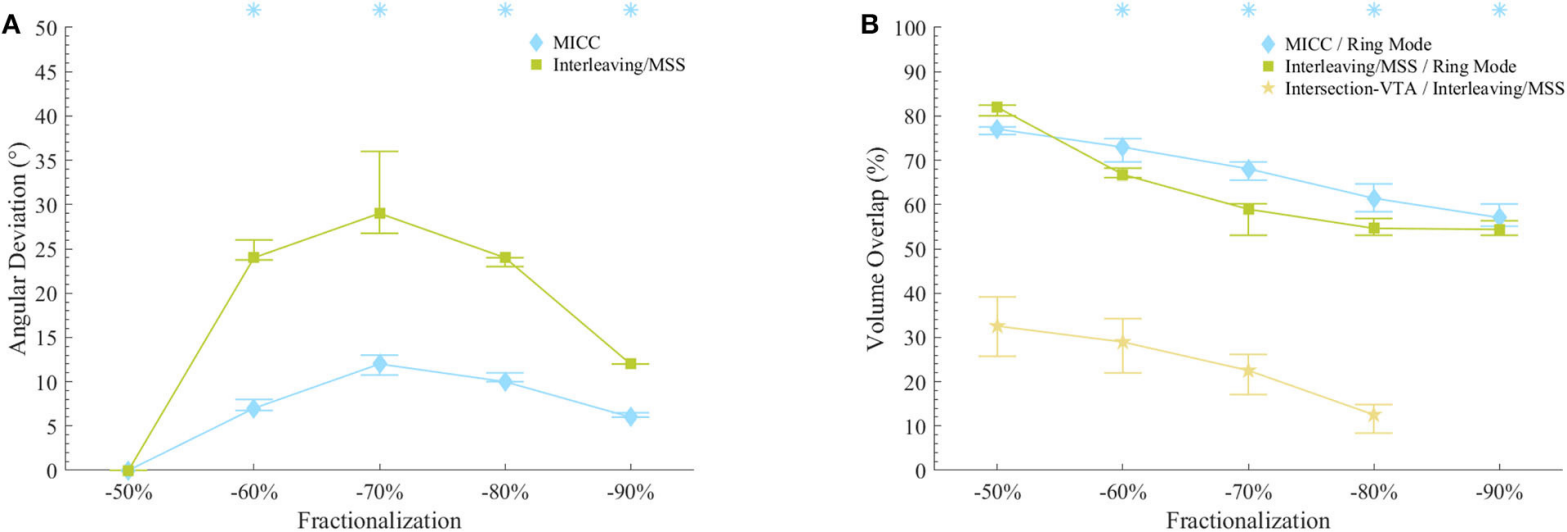
VTAs characterization for fractionalizations. **(A)** VTA deviations from the expected rotation angles for MICC and Interleaving/MSS. Asterisks indicate fractionalizations for which MICC had significantly lower (*p* < 0.05) angle deviations compared to Interleaving/MSS. **(B)** VTA volume overlap ratio for MICC/ring mode, Interleaving/MSS/ring mode and Intersection VTA/Interleaving/MSS. For the fractionalization −90%/−10%, Interleaving/MSS always failed to produce VTA 2 and therefore, there was no Intersection VTA. Asterisks indicate fractionalizations for which MICC had significantly higher (*p* < 0.05) overlap ratio compared to Interleaving/MSS. On both plots, markers indicate the median value across all analyzed VTA radii, whereas bars indicate the (25–75%) IQR. Fractionalization percentages indicate the activation of the dominant electrode (Table 1 E2).

**Figure 4:**
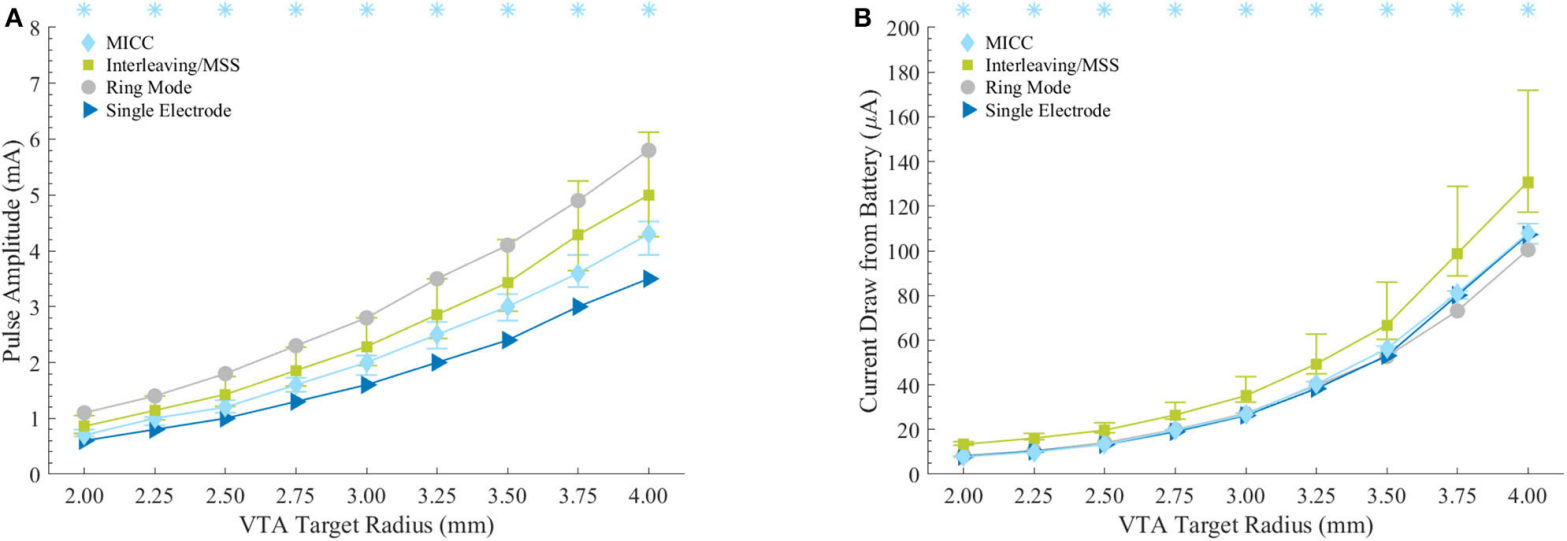
Energy efficiency characterization for the stimulation settings associated to the VTAs. **(A)** Pulse amplitudes necessary to generate the VTAs with defined target radii. Asterisks indicate VTA radii for which MICC needed significantly lower (*p* < 0.05) pulse amplitudes compared to Interleaving/MSS. **(B)** Current draw from battery assuming 3 kΩ as electrode impedance. Asterisks indicate VTA radii for which MICC had significantly lower (*p* < 0.05) current draw from battery compared to Interleaving/MSS. For MICC and Interleaving/MSS on both plots, markers indicate the median value across fractionalization, whereas bars indicate the (25–75%) IQR.

The VTAs for MICC and Interleaving/MSS were characterized based on: (1) their deviation from the expected rotation angle, (2) their volume, (3) the pulse amplitude necessary to generate them, and (4) their associated current draw from battery. This characterization was done for each VTA radius-fractionalization combination (Figures 3, 4). To be consistent with the statistical tests used in this work, results are reported as median (25–75% interquartile range; IQR), except for the current draw from battery scenario with clinically measured electrode impedances, where results are reported as mean ± standard deviation.

For single electrode activation, single source and multiple source current controlled systems generate identical stimulation fields. Moreover, based on Equations (1), (3), and (4), these stimulation fields will have an identical current draw from battery. Therefore, the results reported in this section focus on fractionalizations (i.e., pulse amplitude distributions between two electrodes) unless otherwise noted.

### Characterization of VTAs

The radii for MICC and Interleaving/MSS VTAs showed no significant differences (*p* = 0.17; Supplementary Table 1). For all VTA radius-fractionalization combinations, MICC VTAs showed highly significant lower (*p* < 0.001) deviations from the expected rotation angle (i.e., higher steering accuracy) than Interleaving/MSS VTAs, being 7.0 (4.5–10.5)° and 24.0 (9.0–25.3)°, respectively (Table 2). Across VTA radii, MICC steering accuracy was significantly better (*p* = 0.002) than that of Interleaving/MSS for all fractionalizations excepting −50/−50% (Figure 3A). Steering accuracy for MICC and Interleaving/MSS was perfect for both single electrode activation and the −50/−50% fractionalization, but worsened for intermediate fractionalizations (e.g., −70/−30%, Figure 3A and Table 2). Across VTA radii, the variability (i.e., IQR) of the steering accuracy for each fractionalization was consistent for MICC. In contrast, for Interleaving/MSS this variability was the lowest for fractionalizations of −80/−20% and −90/−10%, and increased for fractionalizations of −60/−40% and −70/−30%, being the highest for the latter.

**Table 2:** VTA deviation from the expected rotation angle in degrees for all fractionalizations.

**Fractionalization**	**–50%/−50%**	**−60%/−40%**	**−70%/−30%**	**−80%/−20%**	**−90%/−10%**
**MICC**	0.0 (0.0–0.0)	7.0 (6.8–8.0)	12.0 (10.8–13.0)	10.0 (10.0–11.0)	6.0 (6.0–6.5)
**Interleaving/MSS**	0.0 (0.0–0.0)	24.0 (23.8–26.0)	29.0 (26.8–36.0)	24.0 (23.0–24.0)	12.0 (12.0–12.0)

*Deviations were calculated based on the expected rotation angles. Medians and (25–75%) IQR were calculated across all VTA target radii*.

The size of VTAs increased for fractionalizations with more evenly distributed pulse amplitudes (Figure 3B and Table 3). MICC VTAs overlapped 67.7 (60.7–74.4)% of ring mode VTAs, whereas for Interleaving/MSS, this overlap was 58 (54.2–68.2)%. For all VTA radius-fractionalization combinations, MICC VTAs were 6.8 (−3.2–11.8)% larger than Interleaving/MSS VTAs, with this difference being highly significant (*p* < 0.001; Table 4). Interleaving/MSS VTAs overlapped MICC VTAs in 89.9 (87.1–95.5)% of their volume. The volume difference between VTAs for MICC and Interleaving/MSS was the highest for fractionalizations of −70/−30%. This phenomenon can be explained by the fact that for this fractionalization, the electrode with 30% of the pulse amplitude failed to generate VTA 2 for some VTA target radii.

**Table 3:** VTA overlap for MICC and Interleaving/MSS for all fractionalizations.

**Fractionalization**	**–50%/–50%**	**–60%/–40%**	**–70%/–30%**	**–80%/–20%**	**–90%/–10%**
**MICC**	77.1 (75.8–77.5)	73.0 (69.6–74.9)	68.1 (65.5–69.6)	61.4 (58.4–64.7)	57.0 (55.1–60.1)
**Interleaving/MSS**	81.9 (80.0–82.5)	66.8 (66.1–68.2)	58.9 (53.1–60.2)	54.6 (53.1–56.9)	54.4 (53.1–56.3)
**Intersection VTA**	32.6 (25.7–39.2)	29.0 (22.0–34.2)	22.5 (17.1–26.2)	12.5 (8.4–14.8)	---

*For MICC and Interleaving/MSS, percentage was calculated based on ring mode VTAs, whereas for Intersection VTA, percentage was calculated based on Interleaving/MSS VTAs. Medians and (25–75%) IQR were calculated across all VTA target radii*.

**Table 4:** Volumes in mm^3^ for all VTA target radii.

**VTA target radius**	**2.00**	**2.25**	**2.50**	**2.75**	**3.00**	**3.25**	**3.50**	**3.75**	**4.00**
**Ring mode**	23.2	32.7	50.6	72.3	94.1	127.9	158.0	199.8	243.6
**Single electrode**	10.9	19.9	27.7	39.9	52.5	69.9	88.4	117.5	142.3
**MICC**	12.9 (12.1–16.1)	23.2 (20.5–25.0)	32.2 (29.5–36.2)	48.4 (44.4–52.4)	65.6 (57.3–69.8)	87.5 (78.0–96.6)	110.4 (100.6–119.6)	140.5 (129.8–155.1)	175.1 (158.4–184.9)
**Interleaving/MSS**	10.9 (10.9–12.9)	19.9 (19.9–26.1)	27.7 (27.7–36.7)	43.2 (39.9–52.9)	57.0 (52.5–68.4)	75.7 (70.1–89.5)	94.7 (89.2–111.3)	124.1 (118.1–144.9)	149.1 (142.7–173.4)
**Intersection VTA**	2.8 (2.8–2.8)	5.2 (3.0–7.3)	9.2 (6.7–11.7)	12.3 (7.2–16.9)	17.8 (11.7–23.7)	21.1 (10.2–31.3)	28.6 (16.1–41.9)	40.8 (24.6–58.9)	51.7 (31.8–74.3)

*For Intersection VTA, only fractionalizations generating two individual VTAs were considered. Medians and (25–75%) IQR were calculated across all fractionalizations*.

For radii below 3.25 mm, Interleaving/MSS was unable to generate two Interleaved VTAs for −70/−30% and −80/−20% fractionalizations, whereas for the −90/−10% fractionalization, Interleaving/MSS failed to generate two Interleaved VTAs for all analyzed radii. This failure to generate two interleaved VTAs indicates a failure to steer radially stimulation fields (Figure 3A). The Intersection VTA had a volume of 26.2 (16.0–32.8)% of the Interleaving/MSS VTA, and it increased for larger Interleaving/MSS VTA radii (Table 4). For each of the VTA target radii, the volume of the Intersection VTA increased linearly for fractionalizations with more equally distributed pulse amplitudes (Supplementary Figures 1E–9E).

### Energy Efficiency of VTAs

Compared to ring mode settings, fractionalizations for MICC required 28.6 (22.7–35.8)% lower pulse amplitudes, whereas this reduction was 18.4 (1.7–29.6)% for Interleaving/MSS. The increase of pulse amplitudes compared to single electrode activation was 23.1 (12.5–31.9)% and 42.9 (25–66.7)% for MICC and Interleaving/MSS, respectively. For all VTA radius-fractionalization combinations, pulse amplitudes were 16.1 (9.2–28.6)% lower for MICC than for Interleaving/MSS, with this difference being highly significant (*p* < 0.001; Figure 4A and Table 5). For both paradigms, pulse amplitudes increased for fractionalizations with more equally distributed pulse amplitudes.

**Table 5:** Pulse amplitudes in mA for all VTA target radii.

**VTA target radius**	**2.00**	**2.25**	**2.50**	**2.75**	**3.00**	**3.25**	**3.50**	**3.75**	**4.00**
**Ring mode**	1.1	1.4	1.8	2.3	2.8	3.5	4.1	4.9	5.8
**Single electrode**	0.6	0.8	1.0	1.3	1.6	2.0	2.4	3.0	3.5
**MICC**	0.7 (0.7–0.8)	1.0 (0.9–1.0)	1.2 (1.1–1.3)	1.6 (1.5–1.7)	2.0 (1.8–2.1)	2.5 (2.3–2.7)	3.0 (2.8–3.2)	3.6 (3.4–3.9)	4.3 (3.9–4.5)
**Interleaving/MSS**	0.9 (0.7–1.1)	1.1 (1.0–1.4)	1.4 (1.2–1.8)	1.9 (1.6–2.3)	2.3 (1.9–2.8)	2.9 (2.4–3.5)	3.4 (2.9–4.2)	4.3 (3.6–5.3)	5.0 (4.3–6.1)

*Medians and (25–75%) IQR were calculated across all fractionalizations*.

**Figure 5:**
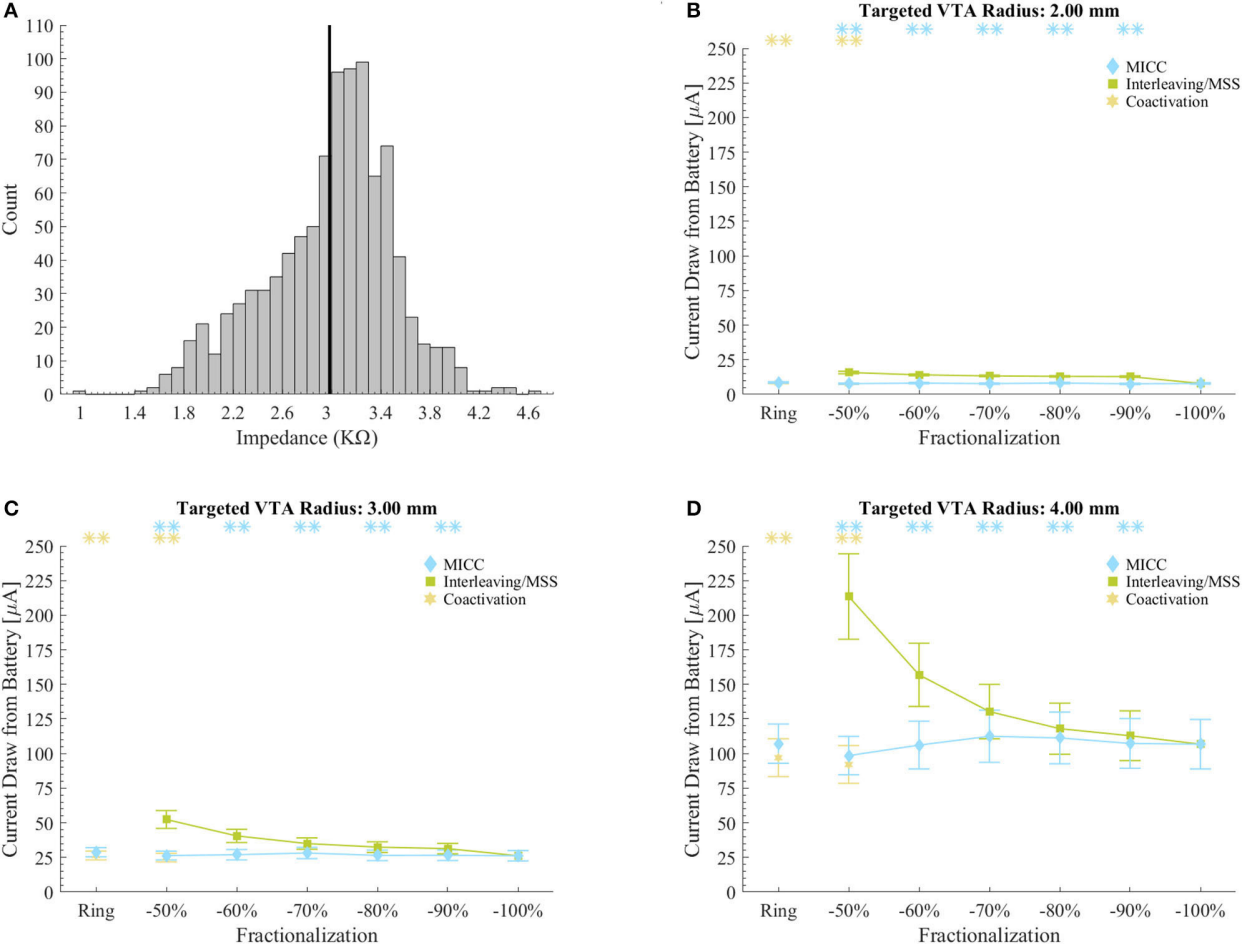
Current draw from battery for clinical impedance measurements. **(A)** Histogram of the impedance values in the set of 980 clinical measurements. The vertical black line shows the mean impedance value. Impedance values were normally distributed (Kolmogorov-Smirnov-test, *p* < 0.001). **(B–D)** Current draw from battery for MICC, Interleaving/MSS and coactivation settings for VTAs radii of 2.00, 3.00, and 4.00 mm. Values were calculated based on the set of 980 impedance measurements. Calculations were done across the possible electrode permutations for the analyzed fractionalizations, where markers indicate the mean and bars indicate ± standard deviation. Asterisks indicate fractionalizations for which MICC had highly significantly lower (*p* < 0.001) current draw from battery compared to Interleaving/MSS (blue) or coactivation had highly significantly lower (*p* < 0.001) current draw from battery compared to MICC (yellow). Fractionalization percentages indicate the activation of the dominant electrode (Table 1 E2).

For the equal electrode impedances scenario and for all VTA radius-fractionalization combinations, MICC had 45.9 (18.8–72.6)% lower current draw from battery than Interleaving/MSS, with this difference being highly significant (*p* < 0.001; Figure 4B and Table 6). Moreover, compared to ring mode and single electrode activation settings, MICC resulted in 1.5 (−5.9–5.2)% lower and 1.3 (−1.9–4.8)% higher current draw from battery, respectively. In contrast, MSS had a current draw from battery increase of 46.3 (23.9–63.3)% and 48.5 (23.6–69.5)%, respectively. For this scenario, an impedance value of 3 kΩ was used to compute current draw from battery for each VTA radius-fractionalization combination. This impedance value was close to the mean impedance value of 2.99 kΩ found for the set of 980 clinical impedance measurements of directional electrodes (Figure 5A).

**Table 6:** Current draw from battery in μA for all VTA target radii.

**VTA target radius**	**2.00**	**2.25**	**2.50**	**2.75**	**3.00**	**3.25**	**3.50**	**3.75**	**4.00**
**Ring mode**	8.3	10.5	14.1	19.9	27.2	39.7	52.7	73.1	100.5
**Single electrode**	7.9	10.2	13.3	19.0	26.3	38.3	53.0	80.1	107.3
**MICC**	7.8 (7.6–8.1)	10.0 (9.9–10.4)	13.3 (13.1–13.5)	19.6 (19.2–19.9)	26.6 (26.2–27.3)	40.3 (38.0–41.5)	56.2 (54.4–57.4)	81.2 (78.5–82.0)	107.9 (103.2–112.2)
**Interleaving/MSS**	13.4 (13.0–14.6)	16.1 (15.4–18.3)	19.7 (18.6–23.0)	26.5 (24.6–32.2)	35.1 (32.3–43.7)	49.4 (44.9–62.7)	66.8 (60.3–86.0)	98.8 (88.8–128.9)	131.0 (117.3–171.9)

*Current draw from battery was calculated considering an electrode impedance of 3 kΩ. Medians and (25–75%) IQR were calculated across all fractionalizations*.

For the scenario with clinically measured electrode impedances and for all VTA radius-fractionalization combinations, MICC had 49.3 ± 35.7% lower current draw from battery than Interleaving/MSS. For all VTA radii and all equal pulse amplitudes distributions (i.e., ring mode and −50/−50% fractionalization), MICC had 6.7 ± 7.7% higher current draw from battery than coactivation. In both cases, the difference in current draw from battery was highly significant (*p* < 0.001). For each of the VTA radii and across pulse amplitude distributions, MICC had low variability in the current draw from battery. In contrast, the current draw from battery for Interleaving/MSS changed dramatically, having consistently higher current draw from battery for fractionalizations with more evenly distributed amplitudes. The slope in the current draw from battery was larger for fractionalizations −60/−40% and −50/−50% (Figures 5B–D and Supplementary Figures 1–9).

## Discussion

Using a theoretical framework, the results of this work suggest that there are substantial differences in the performance of MICC and Interleaving/MSS for the steering of stimulation fields in directional DBS. Both steering paradigms allowed, with some limitations, the radial steering of stimulation fields. However, comparing these two paradigms, MICC shows a higher accuracy and energy efficiency. The comparison of these two paradigms is done based on the characterization of computationally generated VTAs and a model for current draw from battery. Although the clinical validation of these results is out of the scope of this work, they should contribute to clarify the advantages and limitations that these two stimulation paradigms could have in the clinical practice of directional DBS.

### MICC and Interleaving/MSS

MICC involves a multiple current source electronic architecture to activate each electrode in a DBS lead simultaneously and independently. This simultaneous and independent activation allows a controlled and consistent distribution of the pulse amplitude between different electrodes to better shape and steer stimulation fields in DBS. This ability of multiple source systems has also been referred to as Current Steering (10). The results of this work contribute to a growing body of evidence suggesting that in combination with directional electrodes, MICC expands the options to radially steer stimulation fields with a high angular resolution (5, 9).

Interleaving/MSS is a time domain stimulation approach available to commercially available DBS systems. For Interleaving/MSS, single electrodes are activated sequentially and alternatively to stimulate different areas with defined pulse amplitudes (13, 24). The alternate stimulation of different brain structures like the subthalamic nucleus and substantia nigra pars reticulata has been suggested to improve gait disorders in PD patients (25). Moreover, Interleaving at low frequencies has been suggested to treat simultaneously appendicular symptoms as well as gait and speech problems in PD patients (12). Because single source systems lack the necessary electronic architecture to activate each electrode independently, Interleaving/MSS has also been suggested as an alternative for directional leads to radially steer stimulation fields in a similar fashion to MICC by means of a temporal fractionalization (i.e., staggering) of pulse amplitudes (9).

Computationally generated VTAs are binary representations of an electric field crossing the activation threshold of neural tissue. For MICC, simultaneously generated electric fields produce a field result of their sum. Therefore, even low pulse amplitude pulses, in combination with higher pulse amplitudes on the adjacent electrode, will contribute to the shaping of the resulting VTA. In contrast, the electric fields used by Interleaving/MSS will act independently (i.e., they will not produce a field result of their sum) on neural tissue due to their temporal staggering nature. Therefore, for fractionalizations with unequal pulse amplitudes, the electric field produced by an electrode activated with a low pulse amplitude may be insufficient to cross the activation threshold of neural tissue. The mechanisms of action to activate neural tissue for MICC and Interleaving/MSS highlight the fundamental difference between these two stimulation paradigms.

### VTA Modeling

Computationally generated VTAs have been used as an aid to guide, optimize, and simplify the programming of DBS settings (15, 17–19), generate probabilistic stimulation maps to define brain regions with higher probability of producing good clinical outcomes (20, 21), and as in this work, to characterize stimulation fields in DBS under several conditions (9, 22).

In a previous work it was suggested that fractionalizations of pulse amplitudes through different electrodes might need additional amplitude titration in clinical practice (9). This titration was done *in silico* in this work for MICC and Interleaving/MSS by adjusting the total pulse amplitudes for the different fractionalizations to achieve consistent radii across compared VTAs. The choice of generating VTAs with defined target radii is based on the fact that their spatial extent resemble more closely their intended use, which is to estimate the activation of large excitable fibers of passage (e.g., the internal capsule) that are likely to produce stimulation induced side effects (26). Moreover, comparing VTAs with the same radius allows for more appropriate comparisons between MICC and Interleaving/MSS stimulation settings.

In contrast to MICC, Interleaving/MSS requires the generation of two separate VTAs to radially steer stimulation fields. Therefore, this work characterizes both the union and the intersection of Interleaving/MSS VTAs. It is not controversial to expect that different stimulation frequencies may produce different clinical results. However, the complicated nature of temporal staggered stimulation fields makes it difficult to interpret interleaved VTAs in a physiologically consistent manner.

This work also includes the characterization of the VTAs rotation angle, which was done at the cross section at the vertical center of the electrodes generating them. The choice of characterizing only this electrode-center two-dimensional plane is in line with the assumption that the VTA radius is the relevant comparator. A comparison of stimulation paradigms might justifiably use the radius, centroid, volume, or other features of the VTAs as an appropriate control and may also consider different vertical levels. However, a thorough characterization of which measures are best suited for comparing stimulation paradigms is beyond the scope of this work.

Coactivation is included in this work as an additional fractionalization scenario, which involves the simultaneous activation of two or more electrodes in single source current controlled systems. This simultaneous activation aims to distribute pulse amplitudes equally through the activated electrodes. However, the relative impedance between the simultaneously activated electrodes becomes significantly more important for coactivation, as it will determine the shape of the generated VTA. In single source current controlled systems, the simultaneous activation of multiple electrodes would lead to an equal distribution of the pulse amplitude strictly when electrode impedances are equal. Only under this condition, single source current controlled systems and MICC will produce identical VTAs. In contrast, unequal impedances will result in unintentional steering of the VTA toward the electrode with the lowest impedance (5). Therefore, although coactivation may result in less current draw from battery than MICC, it may result in aberrant stimulation fields product of impedance differences between activated electrodes. A comparison of the radial steering accuracy between MICC and coactivation is beyond the scope of this work. Because a current controlled architecture and a homogenous brain model with isotropic tissue conductivity was considered for the generation of VTAs in this work, changes in electrode impedances would not modify the shape of the generated VTAs for MICC or Interleaving/MSS. However, electrode impedances do impact the energy efficiency of these two paradigms.

### Steering Accuracy

The simultaneous and independent activation of electrodes with different pulse amplitudes allowed MICC to radially steer the VTAs for all VTA radius-fractionalization combinations with a median steering accuracy of 7.0°. In contrast, since Interleaving/MSS involves the alternate activation of individual electrodes, the electric field generated by low pulse amplitudes was in several cases not enough to contribute to the shaping of the Interleaving/MSS VTA, leading to a steering accuracy of 24.0°. Although both steering paradigms show a delay to radially steer VTAs, Interleaving/MSS shows a considerable larger delay than MICC, which is consistent with previous reports (9).

Across VTA radii, the variability in the steering accuracy of MICC was consistent for fractionalizations with unequally distributed pulse amplitudes. This low steering accuracy variability suggests that for MICC, there is a systematic deviation from the expected rotation angle of the VTA for all the analyzed fractionalizations. In contrast, for Interleaving/MSS this variability was worst (i.e., largest IQR) for fractionalizations −60/−40% and −70/−30%, which were the fractionalizations that most often succeeded into generating two VTAs, and therefore steered the Interleaving/MSS VTA radially. Although fractionalizations of −80/−20% and −90/−10% showed a lower variability in steering accuracy, these fractionalizations were often unable to steer the VTAs radially due to the failure of Interleaving/MSS in generating a second VTA. This failure resulted in Interleaving/MSS VTAs with a systematic deviation from the intended rotation angle.

Although directional DBS enables more selective activation of neural tissue, this technology also involves new challenges in the implant procedure of the leads and the programming of the stimulation settings (5, 16). A comparison between the actual and intended orientation of implanted directional leads has revealed a deviation of more than 30° in 41% of the analyzed leads (7). Therefore, the accuracy in the steering of stimulation fields play an important role in directional DBS when intending to stimulate specific anatomical structures. A better stimulation field steering capability may compensate for small deviations in the intended implant orientation of directional leads and allow physicians to fully exploit the advantages of directional DBS technology.

### Energy Efficiency

The results of this work show that for all analyzed fractionalizations, both the total pulse amplitude and current draw from battery needed to generate VTAs with a given radius were lower for MICC than for Interleaving/MSS. Moreover, for each of the VTA radii, the current draw from battery was consistent across all analyzed fractionalizations (including the activation of a single electrode and ring mode settings) for MICC. These results demonstrate that activating multiple electrodes simultaneously does not necessarily imply a higher energy demand for MICC. In contrast, the current draw from battery for Interleaving/MSS was always higher when two electrodes were activated, which is in part related to the overhead power needed to activate the electrodes alternatively. It is also worth noting that the current draw for Interleaving/MSS was the lowest for fractionalizations with the largest difference in the electrode activation amplitudes, which were also the fractionalizations that most frequently failed to generate two VTAs.

The occasional failure in the generation of VTAs for Interleaving/MSS has large implications for its energy efficiency in addition to its limitations to radially steer stimulation fields. The energy efficiency for Interleaving/MSS is negatively impacted due to the alternate activation of electrodes (27), which requires an increase in current draw from battery, even if these electrodes are activated with low amplitudes. Additionally, the individual VTAs necessary for Interleaving/MSS produce an Intersection VTA where stimulation would be delivered with an artificially increased stimulation frequency. Although some clinical applications have been suggested for the stimulation frequency heterogeneity in Interleaving/MSS (12), its clinical effects are still poorly understood (24). The results of this work show that the percentage of Interleaving/MSS VTAs that these Intersection VTAs cover, increases for higher VTA target radii and fractionalizations with more evenly distributed pulse amplitudes, which in turn contributes to higher current draw from battery for Interleaving/MSS. Moreover, the size and shape of Intersection VTAs also depend on heterogeneous tissue electrical conductivities, which were not considered for the simulations in this work.

The simultaneous activation of electrodes, together with the lower pulse amplitudes needed and the avoidance of Intersection VTAs most likely contribute to the substantial lower current draw from battery for MICC compared to Interleaving/MSS. The theoretical results of this work suggest that MICC would be a more energy efficient method to generate and accurately steer stimulation fields in directional DBS. A higher energy efficiency in DBS systems is an important aspect, as it may lead to less recharging burden and smaller sizes of rechargeable pulse generators. Moreover, in the case of primary cell systems, higher energy efficiency may lead to less replacement procedures, which have been suggested as having an elevated infection risk compared to the primary implant procedure (28).

### Limitations

The main limitations of this work lie on its theoretical nature and are manifold. The first limitation is the use of a VTA model that specifically considers internal capsule fibers (axons with a diameter of 5.7 μm) that are aligned in a parallel regular grid. The second limitation is the use of a brain model with a homogeneous bulk tissue with fixed isotropic conductivity of 0.2 S/m. The third limitation, and probably the most important, is the unknown clinical relevance of the findings for patients with DBS systems.

Although the modeling of VTAs is an effective tool to visualize the effects of DBS, VTA models have several limitations (16). VTA models constitute a simplified version of Field Cable (FC) models, which are the most detailed models to study the activation of axonal pathways. More advanced FC models include both more realistic orientation and diameter of axons. In addition to internal capsule fibers modeled with a diameter of 5.7 μm, these models may also consider axon diameters of 1.8 μm, which correspond to hyperdirect pathway fibers diameter estimates (29). These added considerations make FC models more technically complex and therefore, computationally more expensive (30).

Comparing the performance of advanced FC models with VTA models similar to the one used in this work, VTA models have a low error in estimating the activation thresholds of internal capsule fibers, whereas they tend to underestimate the thresholds for fibers in the hyperdirect pathway (30). The activation of capsular fibers is suggested to provoke stimulation side effects (26), whereas the activation of hyperdirect fibers has been suggested to be involved in the mediation of voluntary movements (31). The VTA model used in this work constitutes a conservative approach that favors a more reliable estimation of activation thresholds for fibers provoking side effects in order to avoid their stimulation rather than an accurate estimation of the activation of fibers that result in clinical benefits.

More complex VTA models incorporate more detailed aspects involved in DBS, such as heterogeneous properties of brain tissue (32) or both heterogeneous and isotropic properties (33). As in the case of FC models, these heterogeneous brain tissue models could result in more realistic representations of the effect of DBS through more accurate subject specific VTAs. However, this higher accuracy would come at the expense of a higher computational cost.

One potential advantage of using a homogeneous brain model is that generalizing findings based on a single heterogeneous brain tissue model may mask more broadly applicable principles. For instance, it would be expected that for single source systems, the mean orientation angle of VTAs produced from coactivation of multiple electrodes, across different lead placements and electrode rotations, will be deviated toward the electrode with the lowest impedance. However, if a heterogeneous model is used, this effect might be masked for particular electrode orientations that compensate for these impedance differences (9). In these conditions, a homogeneous brain model may be more appropriate for exploring generalizable conclusions, while a heterogeneous and anisotropic brain model would be better suited for inferring conclusions about a single subject.

The generation of a single heterogeneous brain tissue model might involve years of work of a group of experts (32), which presents a non-trivial hurdle for the implementation of more detailed models for a generalized characterization of stimulation fields in clinical DBS. Moreover, averaging the results of several heterogeneous brain tissue models will likely resemble the results that emerge when using homogeneous or semi-homogeneous models. This may explain the persistent usage of homogeneous and semi-homogeneous brain tissue models in studies that intend to explore more generalizable phenomena (20–22).

Although the analyses in this work are focused on two neighboring electrodes on a single vertical level of a directional lead, the principles described here should equally apply to vertical current steering between electrode levels. Vertical current steering to activate regions between electrode levels has demonstrated clinically significant benefits (34–37). More recently, researchers have also begun exploring activation of electrodes with different polarities on the same vertical level (i.e., anodic, bipolar, or semi-bipolar activation) (38) or reversing the electrode activation polarity (39). These new options for stimulation settings hold great promise for future programming techniques, as they present an opportunity to further shape stimulation fields and achieve a more selective stimulation of brain structures.

The results of this work offer a strictly theoretical characterization of the effectiveness, efficacy and limitations of MICC and Interleaving/MSS in the steering of stimulation fields in directional DBS. Therefore, they should be interpreted in the context of the sparse direct evidence in their translation to clinical practice. *In vivo* measurement of tissue activation poses several challenges that makes it prohibitive to perform. A hypothetical clinical study that may validate the findings in this work would correlate the VTA generated for MICC and Interleaving/MSS with the side effect activation thresholds of the directional settings used in this work, which constitute a more thorough directional exploration than previously reported (4, 6). The prediction of activation of fibers leading to clinical benefits or side effects has previously been suggested as clinical validation for VTA models of DBS leads with conventional ring (40–43) and directional electrodes (44, 45). Although these validation efforts have found some correlation between VTA models and clinical effects, their results are still inconclusive, which points out the challenges of clinically validating VTA models.

## Data Availability Statement

The datasets presented in this article are not readily available because of the Boston Scientific Data Sharing Policy. Requests to access the datasets should be directed to Mauricio Juárez-Paz León, leon.juarezpaz@bsci.com.

## Ethics Statement

The studies involving human participants were reviewed and approved by the Local Ethical Boards at all study sites. The patients/participants provided their written informed consent to participate in this study.

## Author Contributions

LJ-P conceptualization, calculations, data analysis, and manuscript preparation.

## Conflict of Interest

LJ-P is a full-time employee of Boston Scientific Corporation.
