# Supplementary material for: In silico Accuracy and Energy Efficiency of Two Steering Paradigms in Directional Deep Brain Stimulation
Source: Front Neurol. 2020 Oct 30;11:593798. doi: 10.3389/fneur.2020.593798 (PMC7661934; doi:10.3389/fneur.2020.593798)
Supplement: Supplementary file 1 [file Data_Sheet_1.pdf]

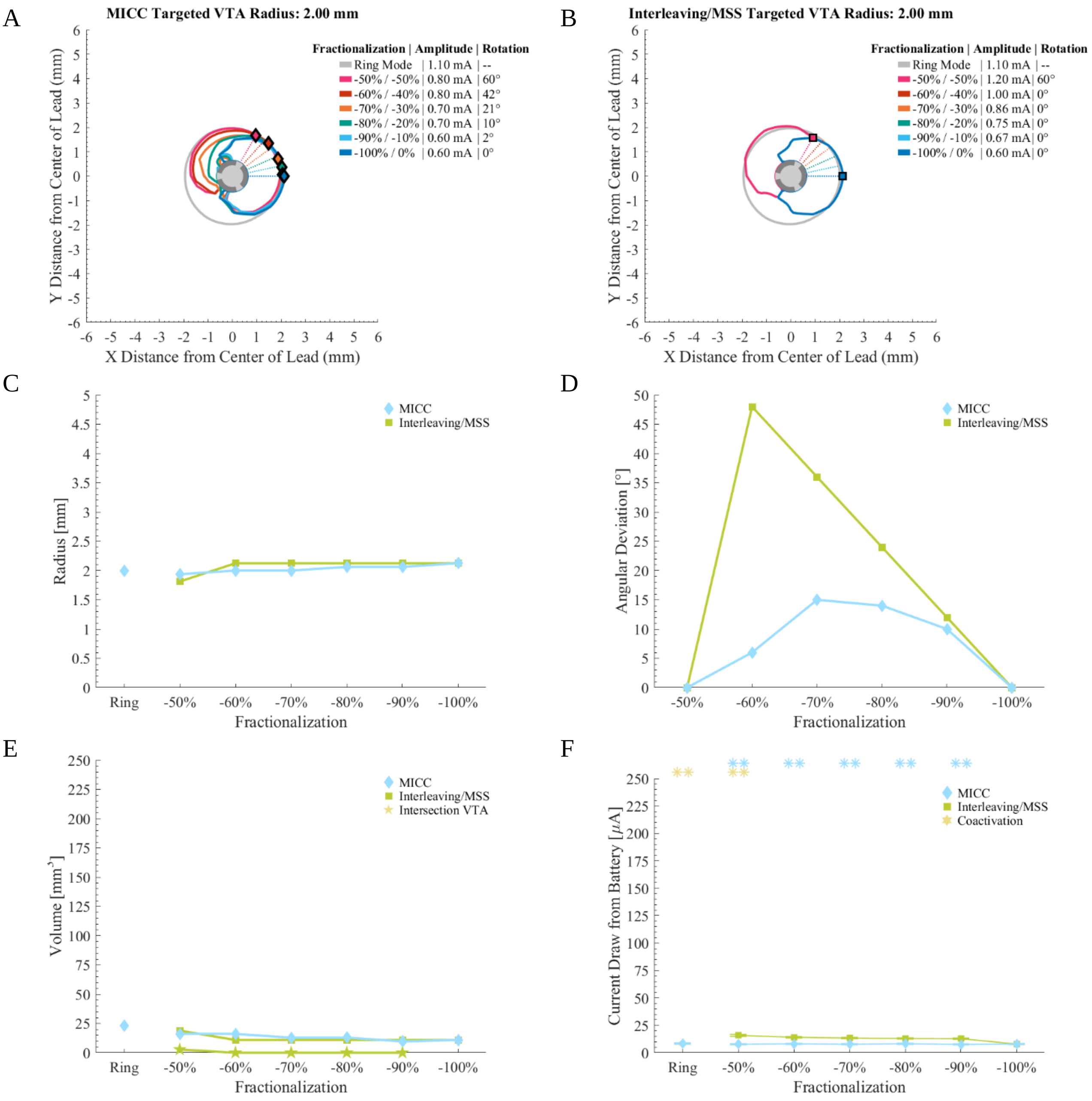

Supplementary Figure 1 | Characterization of VTAs with a target radius of 2.00 mm. **(A)** Cross section of the generated MICC VTAs. **(B)** Cross section of the generated Interleaving/MSS VTAs. The Intersection VTAs are not shown. In both panels dotted lines indicate the expected rotation angles of the VTAs, whereas rhombi or squares indicate the radii of VTAs for MICC and Interleaving/MSS, respectively. **(C)** Radius of the VTAs generated for MICC and Interleaving/MSS. **(D)** Deviation from expected rotation angles of the VTAs for MICC and Interleaving/MSS. **(E)** Volume of the VTAs generated for MICC as well as the generated Interleaving/MSS VTAs and Intersection VTAs. For Intersection VTAs equal to zero, Interleaving/MSS failed to generate two individual VTAs. **(F)** Current draw from battery associated to MICC and Interleaving/MSS settings, whose values were calculated based on a set of 980 impedance measurements. Current draw from battery calculations were done across all possible electrode permutations for all fractionalizations, where markers indicate the mean and bars indicate ± standard deviation. Asterisks indicate fractionalizations for which MICC had highly significant lower ($p < 0.001$) current draw from battery compared to Interleaving/MSS (blue) or coactivation had highly significant lower ($p < 0.001$) current draw from battery compared to MICC (yellow). **(C-F)** Fractionalization percentages indicate the activation of the dominant electrode (**Error! Reference source not found.** E2).

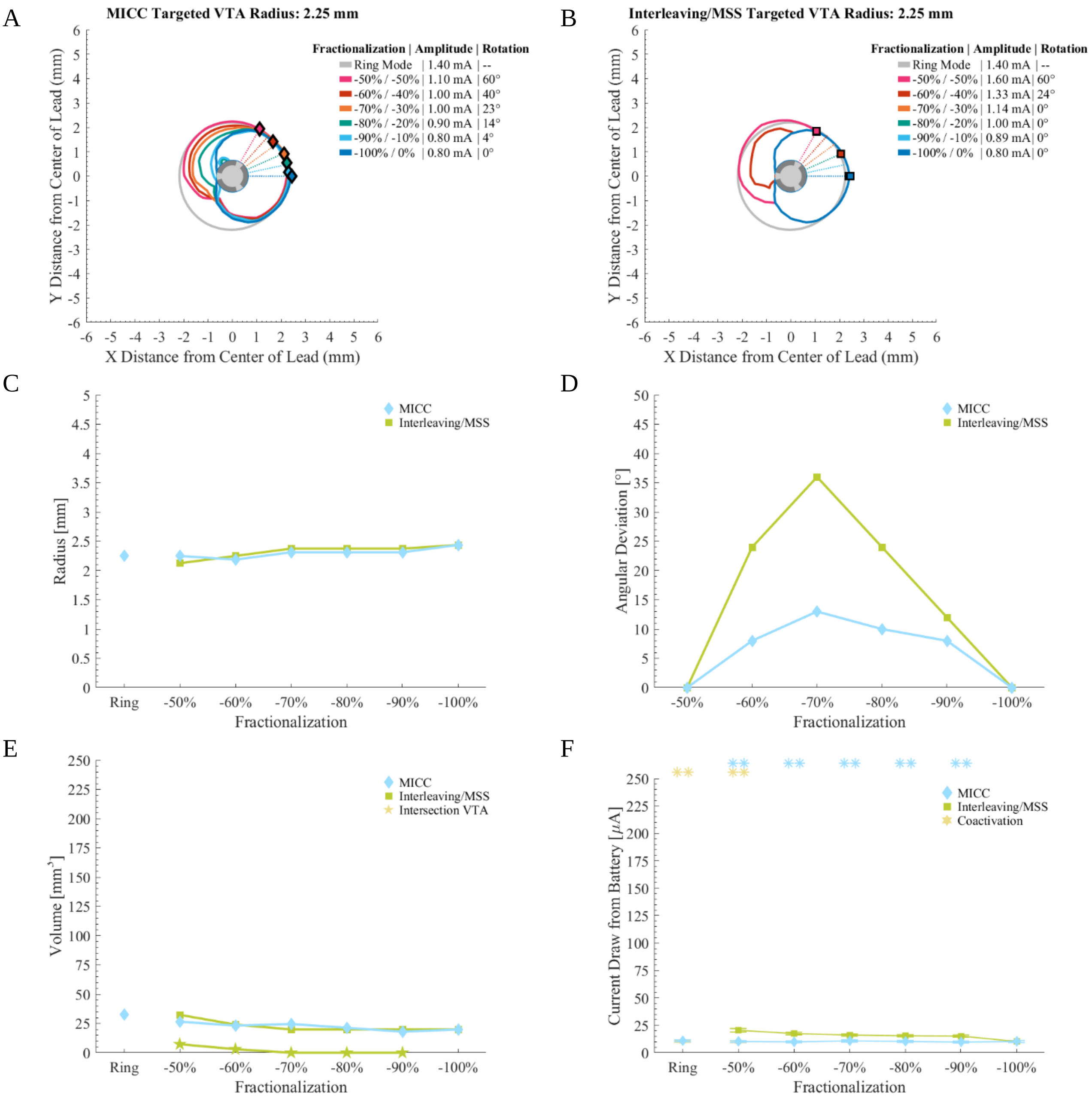

Supplementary Figure 2 | Characterization of VTAs with a target radius of 2.25 mm. **(A)** Cross section of the generated MICC VTAs. **(B)** Cross section of the generated Interleaving/MSS VTAs. The Intersection VTAs are not shown. In both panels dotted lines indicate the expected rotation angles of the VTAs, whereas rhombi or squares indicate the radii of VTAs for MICC and Interleaving/MSS, respectively. **(C)** Radius of the VTAs generated for MICC and Interleaving/MSS. **(D)** Deviation from expected rotation angles of the VTAs for MICC and Interleaving/MSS. **(E)** Volume of the VTAs generated for MICC as well as the generated Interleaving/MSS VTAs and Intersection VTAs. For Intersection VTAs equal to zero, Interleaving/MSS failed to generate two individual VTAs. **(F)** Current draw from battery associated to MICC and Interleaving/MSS settings, whose values were calculated based on a set of 980 impedance measurements. Current draw from battery calculations were done across all possible electrode permutations for all fractionalizations, where markers indicate the mean and bars indicate ± standard deviation. Asterisks indicate fractionalizations for which MICC had highly significant lower ($p < 0.001$) current draw from battery compared to Interleaving/MSS (blue) or coactivation had highly significant lower ($p < 0.001$) current draw from battery compared to MICC (yellow). **(C-F)** Fractionalization percentages indicate the activation of the dominant electrode (**Error! Reference source not found.** E2).

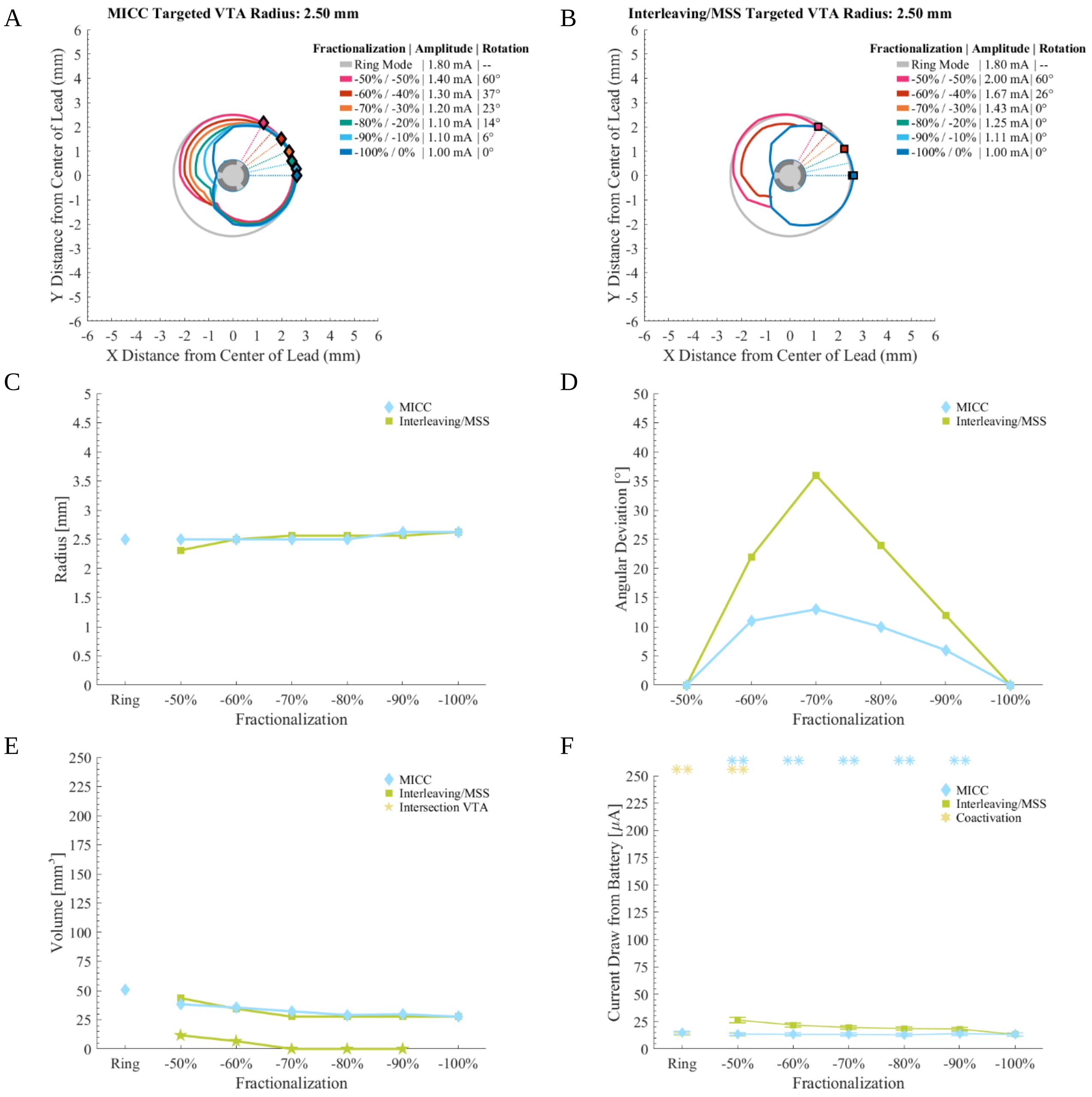

Supplementary Figure 3 **|** Characterization of VTAs with a target radius of 2.50 mm. **(A)** Cross section of the generated MICC VTAs. **(B)** Cross section of the generated Interleaving/MSS VTAs. The Intersection VTAs are not shown. In both panels dotted lines indicate the expected rotation angles of the VTAs, whereas rhombi or squares indicate the radii of VTAs for MICC and Interleaving/MSS, respectively. **(C)** Radius of the VTAs generated for MICC and Interleaving/MSS. **(D)** Deviation from expected rotation angles of the VTAs for MICC and Interleaving/MSS. **(E)** Volume of the VTAs generated for MICC as well as the generated Interleaving/MSS VTAs and Intersection VTAs. For Intersection VTAs equal to zero, Interleaving/MSS failed to generate two individual VTAs. **(F)** Current draw from battery associated to MICC and Interleaving/MSS settings, whose values were calculated based on a set of 980 impedance measurements. Current draw from battery calculations were done across all possible electrode permutations for all fractionalizations, where markers indicate the mean and bars indicate ± standard deviation. Asterisks indicate fractionalizations for which MICC had highly significant lower ($p < 0.001$) current draw from battery compared to Interleaving/MSS (blue) or coactivation had highly significant lower ($p < 0.001$) current draw from battery compared to MICC (yellow). **(C-F)** Fractionalization percentages indicate the activation of the dominant electrode (**Error! Reference source not found.** E2).

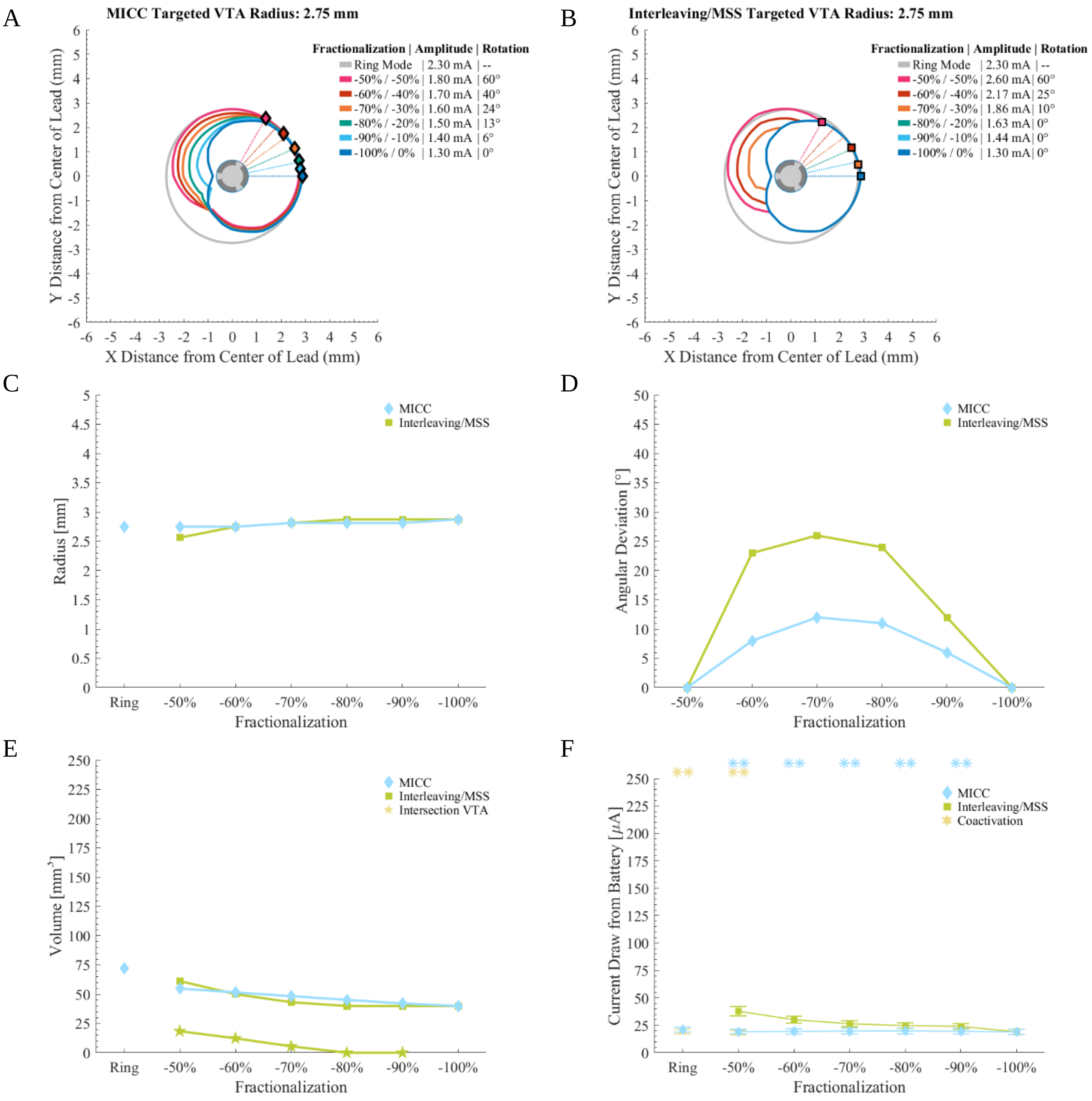

Supplementary Figure 4 | Characterization of VTAs with a target radius of 2.75 mm. **(A)** Cross section of the generated MICC VTAs. **(B)** Cross section of the generated Interleaving/MSS VTAs. The Intersection VTAs are not shown. In both panels dotted lines indicate the expected rotation angles of the VTAs, whereas rhombi or squares indicate the radii of VTAs for MICC and Interleaving/MSS, respectively. **(C)** Radius of the VTAs generated for MICC and Interleaving/MSS. **(D)** Deviation from expected rotation angles of the VTAs for MICC and Interleaving/MSS. **(E)** Volume of the VTAs generated for MICC as well as the generated Interleaving/MSS VTAs and Intersection VTAs. For Intersection VTAs equal to zero, Interleaving/MSS failed to generate two individual VTAs. **(F)** Current draw from battery associated to MICC and Interleaving/MSS settings, whose values were calculated based on a set of 980 impedance measurements. Current draw from battery calculations were done across all possible electrode permutations for all fractionalizations, where markers indicate the mean and bars indicate ± standard deviation. Asterisks indicate fractionalizations for which MICC had highly significant lower ($p < 0.001$) current draw from battery compared to Interleaving/MSS (blue) or coactivation had highly significant lower ($p < 0.001$) current draw from battery compared to MICC (yellow). **(C-F)** Fractionalization percentages indicate the activation of the dominant electrode (**Error! Reference source not found.** E2).

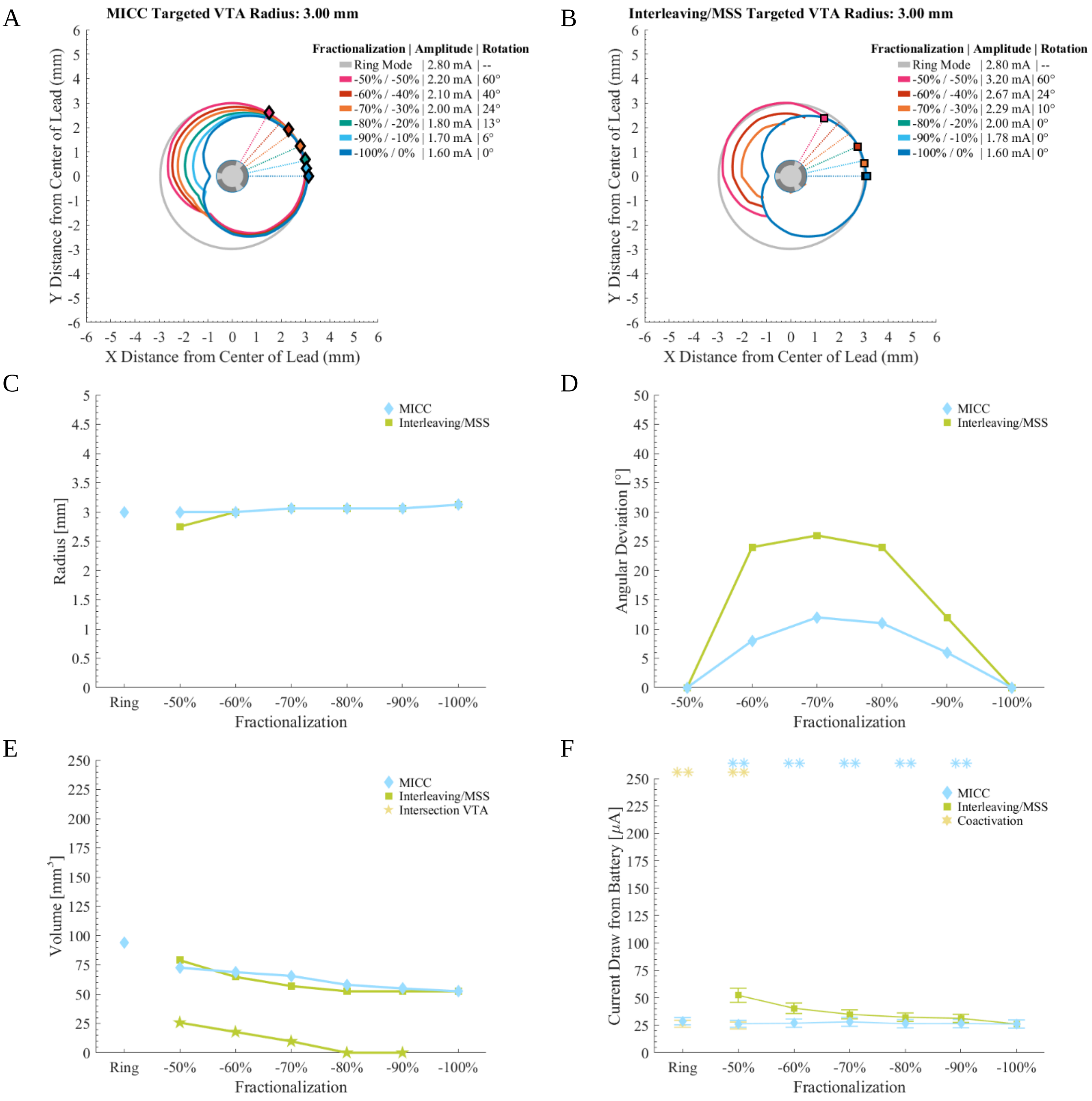

Supplementary Figure 5 | Characterization of VTAs with a target radius of 3.00 mm. **(A)** Cross section of the generated MICC VTAs. **(B)** Cross section of the generated Interleaving/MSS VTAs. The Intersection VTAs are not shown. In both panels dotted lines indicate the expected rotation angles of the VTAs, whereas rhombi or squares indicate the radii of VTAs for MICC and Interleaving/MSS, respectively. **(C)** Radius of the VTAs generated for MICC and Interleaving/MSS. **(D)** Deviation from expected rotation angles of the VTAs for MICC and Interleaving/MSS. **(E)** Volume of the VTAs generated for MICC as well as the generated Interleaving/MSS VTAs and Intersection VTAs. For Intersection VTAs equal to zero, Interleaving/MSS failed to generate two individual VTAs. **(F)** Current draw from battery associated to MICC and Interleaving/MSS settings, whose values were calculated based on a set of 980 impedance measurements. Current draw from battery calculations were done across all possible electrode permutations for all fractionalizations, where markers indicate the mean and bars indicate ± standard deviation. Asterisks indicate fractionalizations for which MICC had highly significant lower ($p < 0.001$) current draw from battery compared to Interleaving/MSS (blue) or coactivation had highly significant lower ($p < 0.001$) current draw from battery compared to MICC (yellow). **(C-F)** Fractionalization percentages indicate the activation of the dominant electrode (**Error! Reference source not found.** E2).

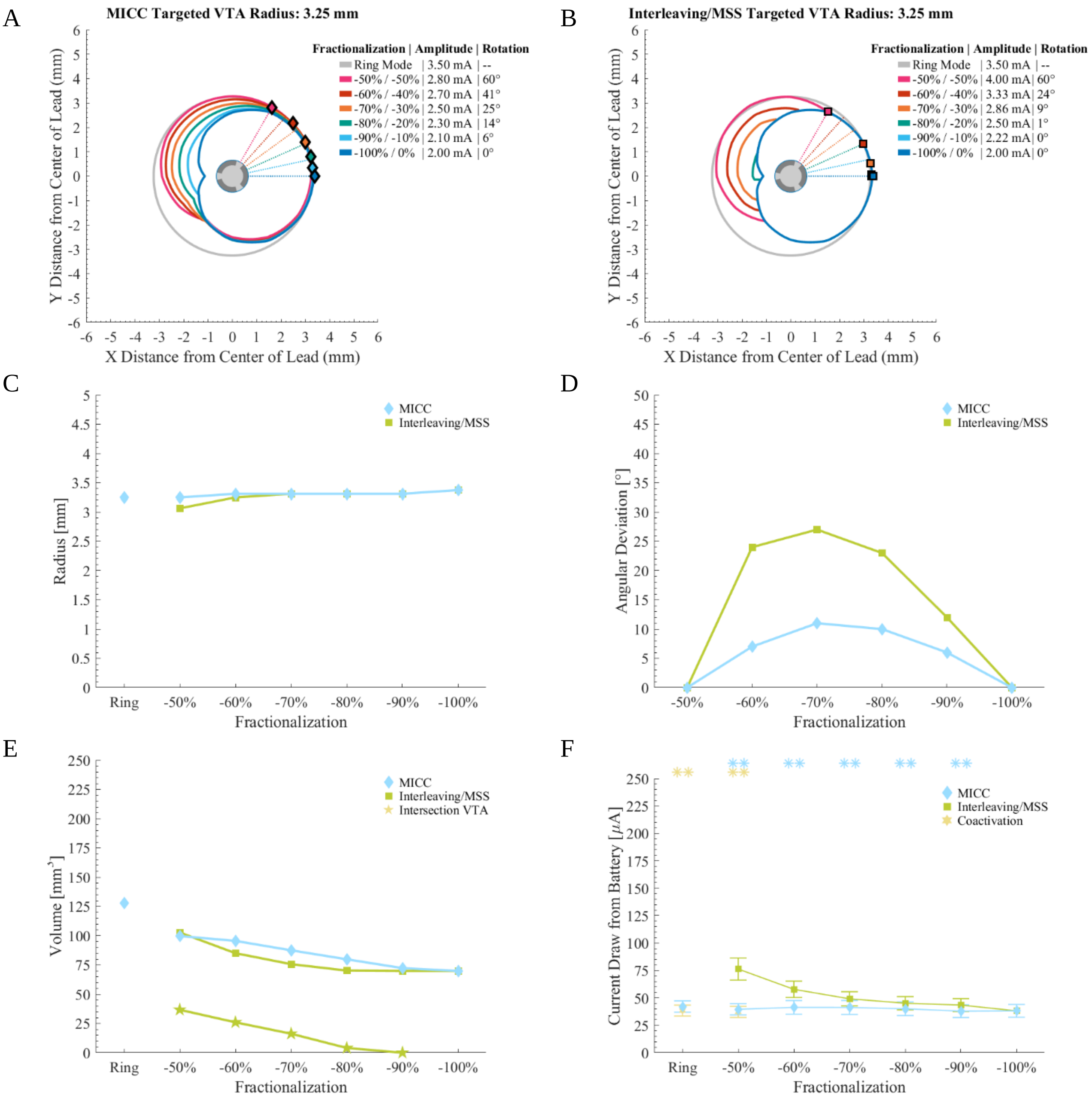

Supplementary Figure 6 | Characterization of VTAs with a target radius of 3.25 mm. **(A)** Cross section of the generated MICC VTAs. **(B)** Cross section of the generated Interleaving/MSS VTAs. The Intersection VTAs are not shown. In both panels dotted lines indicate the expected rotation angles of the VTAs, whereas rhombi or squares indicate the radii of VTAs for MICC and Interleaving/MSS, respectively. **(C)** Radius of the VTAs generated for MICC and Interleaving/MSS. **(D)** Deviation from expected rotation angles of the VTAs for MICC and Interleaving/MSS. **(E)** Volume of the VTAs generated for MICC as well as the generated Interleaving/MSS VTAs and Intersection VTAs. For Intersection VTAs equal to zero, Interleaving/MSS failed to generate two individual VTAs. **(F)** Current draw from battery associated to MICC and Interleaving/MSS settings, whose values were calculated based on a set of 980 impedance measurements. Current draw from battery calculations were done across all possible electrode permutations for all fractionalizations, where markers indicate the mean and bars indicate ± standard deviation. Asterisks indicate fractionalizations for which MICC had highly significant lower ($p < 0.001$) current draw from battery compared to Interleaving/MSS (blue) or coactivation had highly significant lower ($p < 0.001$) current draw from battery compared to MICC (yellow). **(C-F)** Fractionalization percentages indicate the activation of the dominant electrode (**Error! Reference source not found.** E2).

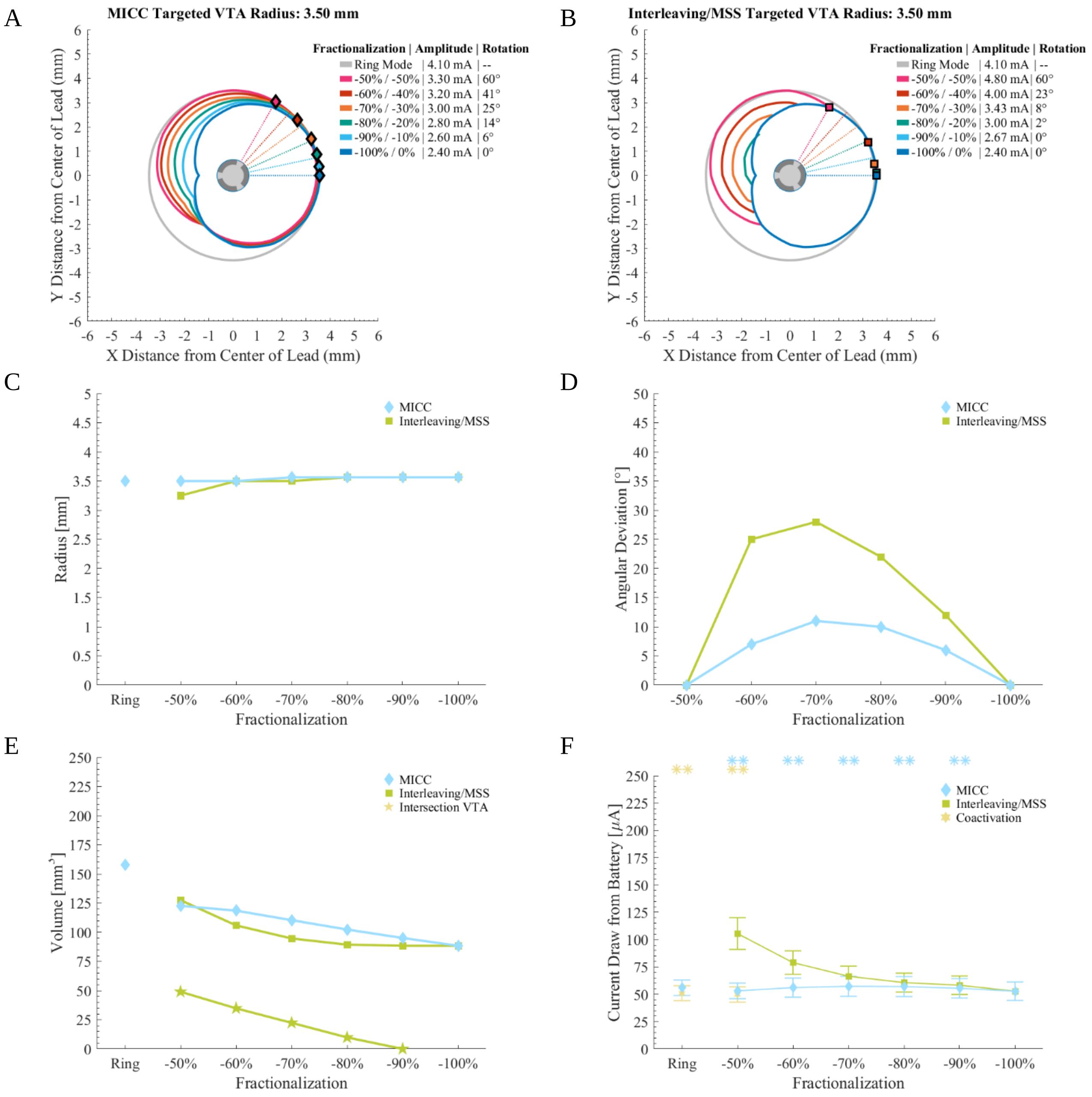

Supplementary Figure 7 | Characterization of VTAs with a target radius of 3.50 mm. **(A)** Cross section of the generated MICC VTAs. **(B)** Cross section of the generated Interleaving/MSS VTAs. The Intersection VTAs are not shown. In both panels dotted lines indicate the expected rotation angles of the VTAs, whereas rhombi or squares indicate the radii of VTAs for MICC and Interleaving/MSS, respectively. **(C)** Radius of the VTAs generated for MICC and Interleaving/MSS. **(D)** Deviation from expected rotation angles of the VTAs for MICC and Interleaving/MSS. **(E)** Volume of the VTAs generated for MICC as well as the generated Interleaving/MSS VTAs and Intersection VTAs. For Intersection VTAs equal to zero, Interleaving/MSS failed to generate two individual VTAs. **(F)** Current draw from battery associated to MICC and Interleaving/MSS settings, whose values were calculated based on a set of 980 impedance measurements. Current draw from battery calculations were done across all possible electrode permutations for all fractionalizations, where markers indicate the mean and bars indicate ± standard deviation. Asterisks indicate fractionalizations for which MICC had highly significant lower ($p < 0.001$) current draw from battery compared to Interleaving/MSS (blue) or coactivation had highly significant lower ($p < 0.001$) current draw from battery compared to MICC (yellow). **(C-F)** Fractionalization percentages indicate the activation of the dominant electrode (**Error! Reference source not found.** E2).

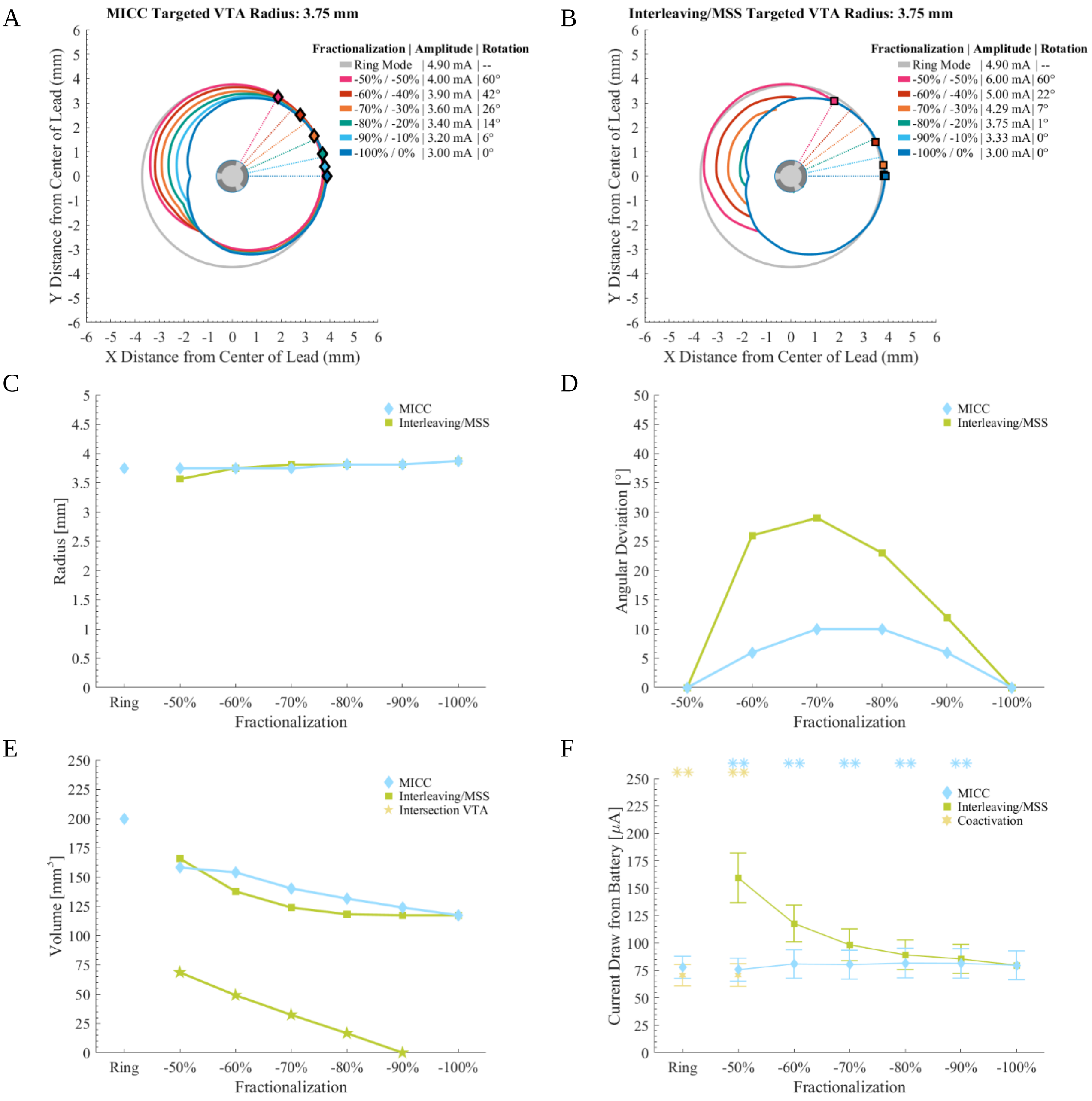

Supplementary Figure 8 | Characterization of VTAs with a target radius of 3.75 mm. **(A)** Cross section of the generated MICC VTAs. **(B)** Cross section of the generated Interleaving/MSS VTAs. The Intersection VTAs are not shown. In both panels dotted lines indicate the expected rotation angles of the VTAs, whereas rhombi or squares indicate the radii of VTAs for MICC and Interleaving/MSS, respectively. **(C)** Radius of the VTAs generated for MICC and Interleaving/MSS. **(D)** Deviation from expected rotation angles of the VTAs for MICC and Interleaving/MSS. **(E)** Volume of the VTAs generated for MICC as well as the generated Interleaving/MSS VTAs and Intersection VTAs. For Intersection VTAs equal to zero, Interleaving/MSS failed to generate two individual VTAs. **(F)** Current draw from battery associated to MICC and Interleaving/MSS settings, whose values were calculated based on a set of 980 impedance measurements. Current draw from battery calculations were done across all possible electrode permutations for all fractionalizations, where markers indicate the mean and bars indicate ± standard deviation. Asterisks indicate fractionalizations for which MICC had highly significant lower ($p < 0.001$) current draw from battery compared to Interleaving/MSS (blue) or coactivation had highly significant lower ($p < 0.001$) current draw from battery compared to MICC (yellow). **(C-F)** Fractionalization percentages indicate the activation of the dominant electrode (**Error! Reference source not found.** E2).

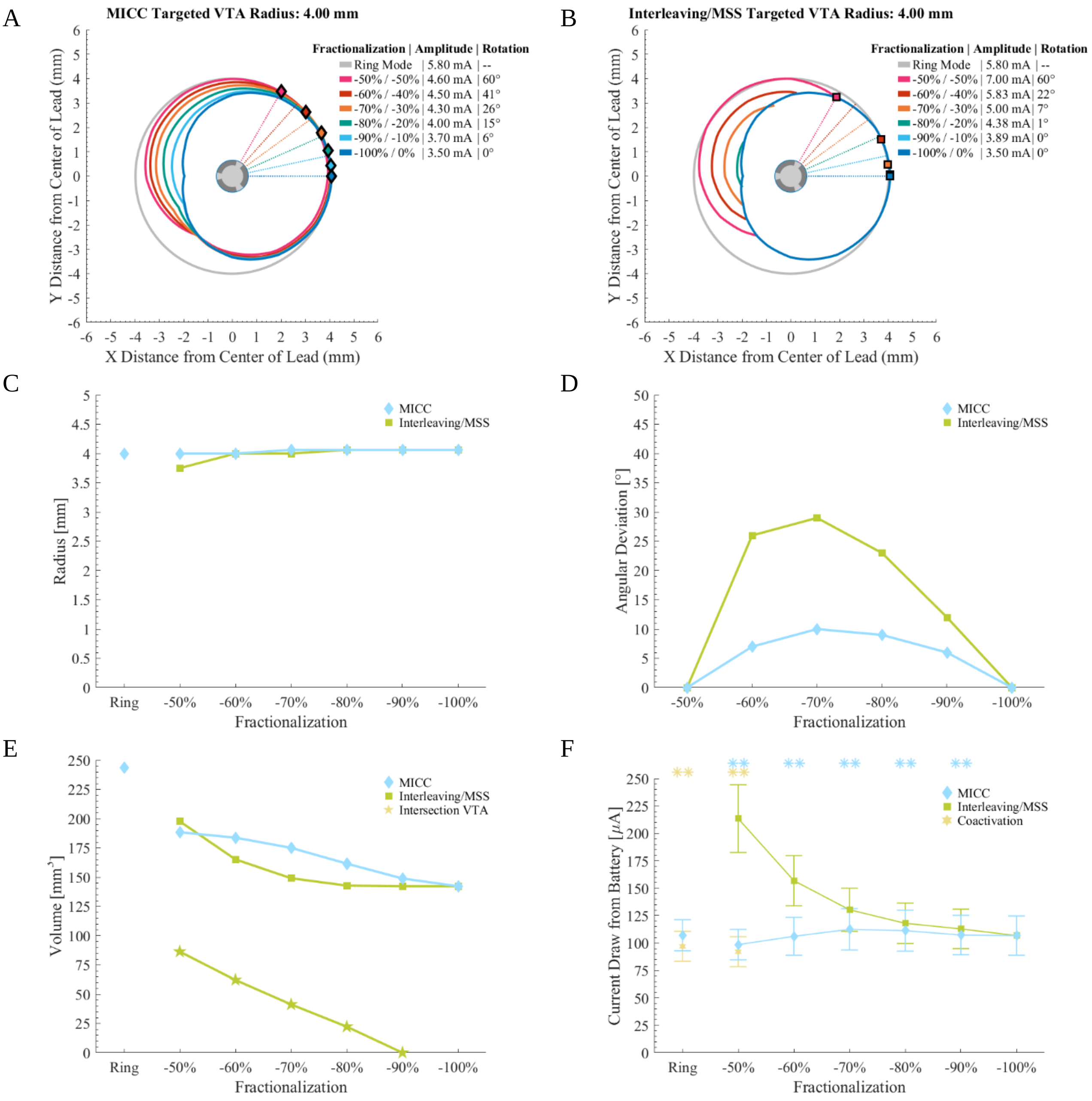

Supplementary Figure 9 | Characterization of VTAs with a target radius of 4.00 mm. **(A)** Cross section of the generated MICC VTAs. **(B)** Cross section of the generated Interleaving/MSS VTAs. The Intersection VTAs are not shown. In both panels dotted lines indicate the expected rotation angles of the VTAs, whereas rhombi or squares indicate the radii of VTAs for MICC and Interleaving/MSS, respectively. **(C)** Radius of the VTAs generated for MICC and Interleaving/MSS. **(D)** Deviation from expected rotation angles of the VTAs for MICC and Interleaving/MSS. **(E)** Volume of the VTAs generated for MICC as well as the generated Interleaving/MSS VTAs and Intersection VTAs. For Intersection VTAs equal to zero, Interleaving/MSS failed to generate two individual VTAs. **(F)** Current draw from battery associated to MICC and Interleaving/MSS settings, whose values were calculated based on a set of 980 impedance measurements. Current draw from battery calculations were done across all possible electrode permutations for all fractionalizations, where markers indicate the mean and bars indicate ± standard deviation. Asterisks indicate fractionalizations for which MICC had highly significant lower ($p < 0.001$) current draw from battery compared to Interleaving/MSS (blue) or coactivation had highly significant lower ($p < 0.001$) current draw from battery compared to MICC (yellow). **(C-F)** Fractionalization percentages indicate the activation of the dominant electrode (**Error! Reference source not found.** E2).

VTA Target Radius	2.00	2.25	2.50	2.75	3.00	3.25	3.50	3.75	4.00
**Ring Mode**	2.00	2.25	2.50	2.75	3.00	3.25	3.50	3.75	4.00
**Single Electrode**	2.13	2.44	2.63	2.88	3.13	3.38	3.56	3.88	4.06
**MICC**	2.00 (1.98 to 2.06))	2.31 (2.23 to 2.31)	2.50 (2.50 to 2.53)	2.81 (2.75 to 2.81)	3.06 (3.00 to 3.06)	3.31 (3.30 to 3.31)	3.56 (3.50 to 3.56)	3.75 (3.75 to 3.81)	4.06 (4.06 to 4.06)
**Interleaving/MSS**	2.13 (2.05 to 2.13))	2.38 (2.22 to 2.38)	2.56 (2.45 to 2.56)	2.81 (2.70 to 2.88)	3.06 (2.94 to 3.06)	3.31 (3.20 to 3.31)	3.50 (3.44 to 3.56)	3.81 (3.70 to 3.81)	4.00 (3.94 to 4.06)

Supplementary Table 1 | Radius in mm for all VTA target radii. Medians and (25% to 75%) IQR were calculated across all fractionalizations
